# Grassland age and local adaptation shape drought resilience across semi‐natural grassland populations

**DOI:** 10.1111/nph.71196

**Published:** 2026-04-22

**Authors:** Yuying Jing, Jenalle L. Eck, Piia Kängsep, Lauri Laaspere, Miina Oras, Laura Puura, Anastasia Tõnisson, Mari Torsus, Martti Vasar, Jianlu Wu, Nianxun Xi, Kadri Koorem, Honor C. Prentice, Marina Semchenko

**Affiliations:** ^1^ Institute of Ecology and Earth Sciences University of Tartu Tartu 50409 Estonia; ^2^ Department of Biology Norwegian University of Science and Technology Trondheim 7491 Norway; ^3^ Institute of Grassland Science Northeast Normal University Changchun 130024 China; ^4^ School of Ecology Hainan University Haikou 570228 China; ^5^ Department of Biology Lund University Lund 22362 Sweden

**Keywords:** carbon fluxes, drought resilience, ecosystem functioning, intraspecific variation, local adaptation, semi‐natural grasslands, soil fungal pathogens, succession

## Abstract

- Increasing frequency and intensity of droughts threaten grassland ecosystems. Semi‐natural grasslands vary in age from ancient to younger sites established on former arable land. While species richness and composition are known to affect drought resilience, little is known about how grassland age shapes drought responses through eco‐evolutionary processes at the plant population level.
- We explored how grassland age and local plant–soil adaptation shape the drought resilience of plant populations by reciprocally combining soils and genotypes of a common grass, *Briza media,* collected from young, intermediate, and ancient grasslands – last cultivated 28–63, 63–84, and > 84–300 years ago, respectively–and subjecting the resulting mesocosms to a drought event.
- Ancient grassland soils enhanced drought resistance and recovery compared with younger soils. Enhanced drought resilience was primarily explained by lower abundance of putative fungal pathogens in older soils. Plants grown in ‘home’ soils from their sites of origin were more productive and invested less in root production to withstand water stress, indicating the important role of local plant–soil adaptation.
- Our results show the long‐lasting legacy of land use history in soil microbial communities and their significant role in shaping drought resilience across grassland populations.

## Graphical abstract

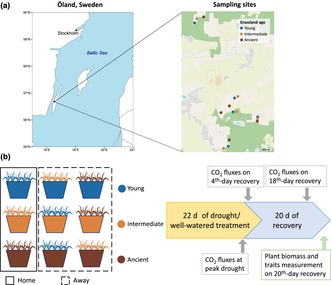

## Introduction

With droughts projected to increase in both frequency and intensity (IPCC, 2023), understanding what makes an ecosystem resilient (defined as the ability to resist and recover from drought) and potential trade‐offs with other ecosystem functions has become a pressing concern (Knapp *et al*., 2024). Semi‐natural grasslands are important for food production and other ecosystem services but are strongly affected by drought (Lyons *et al*., 2023). Ancient semi‐natural grasslands, shaped by centuries of continuous grazing management, are unique for their high plant species richness (Kull & Zobel, 1991; Feurdean *et al*., 2018), which is known to enhance resistance of grassland productivity to drought (Isbell *et al*., 2015). However, most contemporary semi‐natural grasslands have experienced historical land‐use changes, notably conversion to arable land through ploughing, resulting in significant biodiversity loss (Cousins & Eriksson, 2008). The re‐establishment of grassland on former arable land is a slow process, with several decades of restoration still failing to recover plant communities comparable to those in ancient grasslands (Fagan *et al*., 2008). While shifts in plant species composition along grassland successional gradients are well‐documented (Purschke *et al*., 2013; Löfgren *et al*., 2020), population‐level variation in plant traits and local adaptation have been largely overlooked despite their importance in shaping species' adaptive potential under environmental change (Johnson *et al*., 2015). Moreover, although changes in soil conditions and microbial communities have been observed in early‐successional grasslands (Hannula *et al*., 2017; Löfgren *et al*., 2020), the role of plant–soil interactions and local evolutionary processes in modulating drought resilience along the arable‐to‐grassland succession over longer time frames remains unexplored.

As grasslands reassemble on formerly arable land, soil conditions typically transition from nutrient‐rich to nutrient‐poor due to cessation of fertilization and biomass removal by herbivores (Löfgren *et al*., 2020). This edaphic transition is accompanied by a shift from early‐successional plant species with fast resource‐acquisition strategies to late‐successional species exhibiting more resource‐conservative traits (Mudrák *et al*., 2023). Slow‐growing, resource‐conservative species are considered to be more drought‐tolerant (Reich, 2014), suggesting a fundamental trade‐off between growth and stress tolerance across species (Stearns, 1989). Several studies have shown that early‐successional or intensively managed grasslands are less drought resistant compared with older, traditionally managed grasslands, with reduced resistance linked to lower species richness and the dominance of fast‐growing species in the former grasslands (Tilman & Downing, 1994; Oram *et al*., 2023; Korell *et al*., 2024). However, in addition to interspecific trait shifts, intraspecific trait variation, including both phenotypic plasticity and genetic variation, can be substantial (Albert *et al*., 2010) and contribute significantly to total trait variation between communities (Siefert *et al*., 2015) and to differences in drought resilience (Lüscher *et al*., 2022). For example, drought resilience has been shown to vary widely across plant genotypes from distinct climate conditions (Johnson *et al*., 2015; Bristiel *et al*., 2018). However, how traits within a species vary along grassland age gradients and respond to drought events remains unexplored. Furthermore, although the association between resource‐conservative strategy (e.g. high leaf dry matter content, LDMC) and drought resilience has been observed across species (Fernández & Reynolds, 2000; Májeková *et al*., 2021; Mount *et al*., 2024), evidence for the trade‐off between growth potential and drought resilience within species is very limited (Bristiel *et al*., 2018).

Soil microbial communities are also likely to change as soil conditions and plant traits shift along grassland age gradients (de Vries *et al*., 2007; Semchenko *et al*., 2018). Increasing grassland age has been associated with a shift in microbial communities from bacterial to fungal dominance (van der Wal *et al*., 2006; Maharning *et al*., 2009), alongside a transition from predominantly pathogenic to beneficial symbiotic taxa (Hannula *et al*., 2017). Shifts in soil microbial communities, particularly from pathogen‐dominated to mutualist‐dominated communities, can significantly influence grassland responses to drought. In particular, arbuscular mycorrhizal (AM) fungi have been shown to enhance the resilience of grassland productivity under drought conditions (Li *et al*., 2019; Jia *et al*., 2021), while pathogens may reduce drought resilience (Ramegowda & Senthil‐Kumar, 2015). Moreover, local co‐adaptation between plant populations and soil microbial communities may enhance drought resilience. For example, plant genotypes paired with sympatric soil microbes have shown superior performance under drought compared with allopatric combinations when plants and soil communities originated from sites with different water availability (Remke *et al*., 2022). Therefore, changes in soil microbial community composition and local adaptation between plant populations and the soil microbiome may contribute to enhanced drought resilience in older grasslands.

Here, we used a 300‐year grassland successional gradient on the island of Öland (Sweden) and a space‐for‐time approach to assess how eco‐evolutionary shifts in plant traits and plant–soil interactions influence plant population responses to drought. We collected soil and genotypes of *Briza media*, a common grass species, from 15 grassland sites spanning three age groups – young (converted from arable land to grazed grassland 28–63 years ago), intermediate (converted 63–84 years ago), and ancient grasslands (continuously grazed for 84 to over 300 years). We then reciprocally combined the soil and plant genotypes from each grassland age group in a mesocosm experiment under common garden conditions, creating ‘home’ and ‘away’ plant–soil combinations. Following establishment, the mesocosms were subjected to a simulated drought followed by a period of recovery. We assessed drought responses by measuring mesocosm CO_2_ fluxes, above‐ and belowground productivity, and plant traits.

We predicted that ancient grassland soils and plant populations would be more resilient to drought than to younger grasslands, as indicated by higher resistance of carbon fluxes to drought stress and faster recovery, along with sustained aboveground productivity. This expectation is based on predictions that (1) ancient grasslands host more beneficial soil microbial communities and foster more mutualistic, locally adapted plant–soil interactions than younger grasslands, (2) plant genotypes from ancient grasslands exhibit more resource‐conservative traits than those from younger grasslands, and (3) slow‐growing, resource‐conservative genotypes are more drought resilient due to an intraspecific trade‐off between competitive growth under benign, moist conditions and performance under drought. To test the trade‐off, low plant shoot mass and high LDMC under control (moist) conditions were used as indicators of a slow, resource‐conservative strategy, and drought resilience was quantified as the ratio of shoot mass in the drought treatment to that under control conditions.

## Materials and Methods

### Study sites and field sampling

**Fig. 1:**
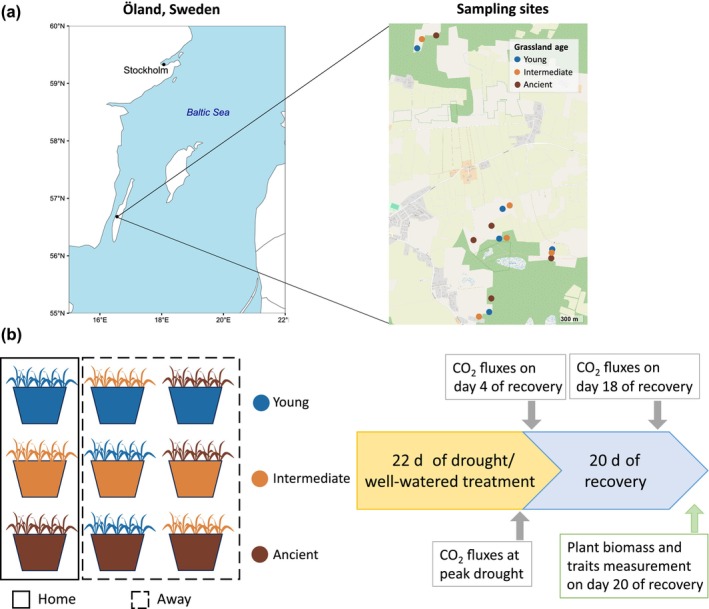
Field sampling sites and mesocosm experimental design. (a) Our study comprised 15 sites representing five replicates of three age groups of grasslands succession from arable land: young (last arable 28–63 years ago), intermediate (last arable 63–84 years ago), and ancient (> 292 years except one site cultivated 84–172 years ago) on the Baltic island of Öland (Sweden). Plant individuals (representing different genotypes of *Briza media*) and soil were collected from each site. (b) In a common garden experiment, clones propagated from the field‐collected plants were grown either in ‘home’ soil (i.e. soil collected from their original sampling site) or in ‘away’ soil (i.e. soil collected from an adjacent grassland of each of the two other age groups). Ten genotypes were planted in each mesocosm. Mesocosms were set up in pairs with identical soil and plant genotype combination. Within each pair of pots, one was subjected to a 22‐day drought treatment followed by a 20‐d recovery period, while the other was maintained under well‐watered conditions throughout the experiment. Ecosystem CO_2_ fluxes were measured at the peak of drought, on the 4th and the 18th day of recovery from drought. Plant traits and above‐ and belowground productivity were determined after 20 d of recovery from drought.

The study comprised 15 grazed semi‐natural grasslands located within a 2 × 4 km area on the island of Öland, Sweden (56°40′–56°42′ N, 16°32′–16°34′ E; Fig. 1a). Grassland age, defined as the continuity of grazing management, was derived from previous reconstructions of land‐use history in the study area based on historical cadastral maps (1723–1797 and 1801–1846), aerial photographs (1938, 1959, and 1994), and field surveys (since 1997) (Johansson *et al*., 2008; Schmid *et al*., 2017). In these earlier studies, grassland age was estimated as an interval, representing the time between the last documented arable use and the first subsequent observation as grassland. We expressed these interval‐based age estimates relative to the 2022 sampling year and classified the grasslands into three age groups based on the period in which they first appeared as grasslands: young (appearing between 1959 and 1994; 28–63 years since arable use), intermediate (1938–1959; 63–84 years), and ancient (present as grasslands for at least 300 years and continuously grazed thereafter, with one site experiencing a temporary arable phase 84–172 years ago) (Schmid *et al*., 2017). Previous studies have shown that young grasslands supported only a few typical grassland specialist species compared with intermediate and ancient grasslands, whereas the species composition of intermediate grasslands was beginning to resemble that of the ancient grasslands (Löfgren *et al*., 2020). We did not include grasslands younger than 28 years, as our model species *B. media* has a low probability of establishing in such grasslands. Each age group was represented by five sites, selected to ensure even spatial distribution (Fig. 1a). All grasslands re‐established naturally following the cessation of crop cultivation, without any reseeding, and have since been managed by grazing. The study area has a mean annual temperature of 7°C and an average annual precipitation of 468 mm (Johansson *et al*., 2008).

We selected *Briza media* L. as our focal plant species because it is commonly present in both early‐ and late‐successional grasslands in this study system and exhibits genetic variation between populations from different successional stages (Prentice *et al*., 2006). In September 2022, at each of the 15 sites, 20 individuals of *B. media* were collected, each excavated as a 5 × 5 cm soil plug that included surrounding plant species. Plugs were taken at least 2 m apart, a distance sufficient to ensure that each individual represented a unique genotype (300 genotypes in total), as the study species is self‐incompatible, wind‐pollinated, and exhibits limited clonal dispersal (Dixon, 2002). After extracting *B. media* genotypes from the soil plugs, we preserved the remaining native vegetation together with the soil in four 1‐L pots per site to maintain fresh soil inocula for the subsequent mesocosm drought experiment.

At the time of plant collection, we also collected topsoil (0–20 cm) from each site for the characterization and for use in the mesocosm experiment. We characterized the soil for texture, pH, total organic carbon, total nitrogen, ammonium‐N, nitrate‐N, Olsen‐phosphorus, and total phosphorus (see Supporting Information Methods S1, for further detail).

To generate clones from the field‐collected *B. media* genotypes, we transplanted individual cuttings of each genotype (*c*. 3 cm in size with intact shoot and root buds) into 200‐mL pots filled with a sterile sand and soil mixture (N% 0.51, C% 6.09, pH 7.0) and propagated them under common glasshouse conditions (20°C, additional light of 50 μmol m^−2^ s^−1^ for 16 h d^−1^, daily watering) for 7 months before using newly propagated cuttings in the mesocosm drought experiment.

### Mesocosm drought experiment

We factorially combined *B. media* populations (represented by 10 randomly selected genotypes from each grassland site) and soils in ‘home’ (plants and soil from the same site) and ‘away’ (plants with soil from an adjacent grassland of different age group) plant–soil combinations and subjected them to a drought treatment (Fig. 1b). The experiment included 90 mesocosms in total: 3 grassland age groups × 5 replicate sites per age group × 3 plant–soil combinations (one ‘home’ and two ‘away’) × 2 treatments (control and drought).

In May 2023, we thoroughly mixed soil collected from each site and distributed it into 2‐L pots (13 cm lower diameter, 15.5 cm upper diameter, 13 cm height), with 1.8 kg of field‐collected soil per pot. As *B. media* is a slow‐growing grass (van der Werf *et al*., 1993) and our study sites are characterized by shallow soils (Rosén & Bakker, 2005), the pot volume of 2 L was considered appropriate to accommodate root development under our experimental conditions. To better approximate field microbial communities, we added 200 g of soil from the preserved local plant communities to each pot as an additional source of site‐specific microbial inoculum. From each propagated genotype of *B. media*, we selected individual cuttings with intact shoot and root buds, representing cloned ramets of similar size, and planted them in a full‐factorial design of soil origin (‘home’ vs ‘away’) and drought treatment (control vs drought). We planted the 10 genotypes from the same site into each mesocosm in a regular pattern, spacing adjacent plants 4.5 cm apart. To aid establishment, we kept the plants in a glasshouse for the first month with 16 h of supplemental light (350 μmol m^−2^ s^−1^) at 20°C : 14°C (day : night). In June 2023, we moved the mesocosms to the open‐air garden. Few seedlings of other species emerged from the soil seed bank during the experiment and were removed to maintain planted genotypes of *B. media* only. When setting up the experiment, we dried 10 g of mixed soil from each site using silica gel for fungal community composition analysis via amplicon sequencing (see ‘Molecular identification of soil fungal communities’).

After 3 months of plant growth, we initiated a simulated drought event. To exclude ambient rainfall, we placed all mesocosms in a glasshouse. Before drought initiation, all mesocosms were watered to 100% water‐holding capacity (WHC). During the drought treatment period, we maintained control mesocosms at 60% WHC, the optimal level for plant growth and microbial activity, corresponding to a volumetric soil water content (SWC) of 28–42%. In drought‐treated mesocosms, we applied a gradual drought treatment, increasing severity over three wk to simulate field‐like drought progression. During the first week, no water was supplied, resulting in a decline in SWC to *c*. 20% by day 8. During the second week, we applied a small amount of water to maintain SWC *c*. 10–20% (*c*. 20% WHC). During the third week, no water was applied to intensify the drought. By the end of the drought treatment (day 22; peak drought), SWC declined to 3–8%, and plants showed visible wilting and leaf senescence (Fig. S1). Following the drought period, we rewatered all mesocosms to 100% WHC and moved them to an open‐air garden where they recovered under ambient rainfall and regular watering. We chose the drought intensity and duration based on previous studies showing that soil moisture below 20% WHC for 3 wk markedly reduces plant growth, carbon fixation, and soil microbial activity (de Vries *et al*., 2012; Oram *et al*., 2023; Knight *et al*., 2024).

We measured CO_2_ fluxes in each mesocosm at three time points (at peak drought, after 4 and 18 d of recovery) using EGM‐5 portable CO_2_ gas analyzers (PP Systems, Amesbury, MA, USA). Net ecosystem exchange (NEE) was measured using a transparent chamber (19.5 × 20 × 30 cm, L × W × H) under natural light conditions, followed by measurements of ecosystem respiration using an identical opaque chamber. Each measurement lasted 120 s and was conducted under clear‐sky conditions during midday hours to ensure relatively high and comparable illumination among mesocosms. The rate of CO_2_ concentration change was determined from the linear range of the measurement curve, identified by visual inspection and following Pirk *et al*. (2016). NEE and ecosystem respiration (g m^−2^ h^−1^) were calculated using the following equation:

$$F_{{\mathrm{CO}}_{2}}=\frac{d{\mathrm{CO}}_{2}}{\mathrm{dt}}\times \frac{P}{1013}\times \frac{273}{273+T}\times \frac{1}{22.414}\times \frac{V}{A}\times 10^{3}\times \frac{1}{6.312}$$

where $F_{{\mathrm{CO}}_{2}}$ is the CO_2_ flux (g m^−2^ h^−1^), $d{\mathrm{CO}}_{2}/\mathrm{dt}$ is the linear rate of change in CO_2_ concentration (μmol mol^−1^ s^−1^), $\frac{P}{1013}$ is the correction for barometric pressure with *P* measured in mbar, $\frac{273}{273+T}$ is the correction for air temperature with *T* input in °C, $\frac{1}{22.414}\;\left(\mathrm{mol}\;l^{-1}\right)$ is the molar volume of an ideal gas at standard temperature and pressure, *V* is the chamber air volume (0.010159 m^3^) and *A* is the soil surface area (0.0181 m^2^), and $\frac{1}{6.312}$ is the unit conversion factor from μmol m^−2^ s^−1^ to g m^−2^ h^−1^.

The sign of NEE was reversed so that positive values indicate net CO_2_ uptake and negative values indicate net CO_2_ emission. Photosynthetic rate, corresponding to gross primary productivity, was calculated as the sum of ecosystem respiration and NEE. Mesocosm measurements were conducted in a randomized temporal sequence, with photosynthetically active radiation (PAR) and atmospheric temperature recorded during each measurement.

On day 20 of drought recovery, we harvested the plants and measured plant traits. We collected aboveground biomass for each genotype and extracted a soil core (diameter 3.8 cm, depth 9 cm) from the center of each mesocosm, positioned between rooting points. We then measured maximum shoot height, aboveground biomass, and LDMC for each genotype (Methods S2). We randomly sampled one leaf from each genotype and pooled the samples by mesocosm for analysis of leaf carbon and nitrogen content using an Elemental Analyzer (FlashEA1112; Thermo Fisher Scientific Inc., Waltham, MA, USA). We retrieved roots from the soil core and scanned them using an Epson Perfection V800 Photo Scanner (Model J221B; Seiko Epson Corporation, Nagano, Japan). Root length, root volume, and average diameter were calculated using RhizoVision Explorer 2.0.3 (Seethepalli *et al*., 2021). We oven‐dried all leaf and root samples at 40°C to constant weight to determine dry biomass. Root traits were calculated as follows: specific root length (SRL; total scanned root length/scanned root dry mass) and root tissue density (RTD; scanned root dry mass/scanned root volume). We estimated total root biomass in each mesocosm by scaling root biomass from the soil core, using the ratio of total mesocosm soil volume to soil core volume. Root mass fraction was calculated as the ratio of root dry mass to total plant dry mass. AM fungal root colonization rate was determined following the ink and vinegar method for root staining (Vierheilig *et al*., 1998) and by recording the presence of AM fungal structures (hyphae, arbuscules, and vesicles) in 100 randomly selected locations within stained root fragments at 100× magnification (McGonigle *et al*., 1990).

### Molecular identification of soil fungal communities

Soil DNA was extracted using the QIAGEN PowerMax Soil DNA Isolation Kit (Qiagen GmbH, Hilden, Germany) from a 5 g subsample of soil, which was collected from each site and used for setting up the drought experiment. To characterize the soil fungal community composition, we amplified the internal transcribed spacer (ITS2) region of the ribosomal RNA gene cluster using the degenerate primer pair fITS7:fiTS7o (Ihrmark *et al*., 2012) and ITS4 (White, 1990). The amplified ribosomal ITS2 regions were sequenced by Novogene Company (UK) using a 2 × 250 bp paired‐read sequencing approach on an Illumina NovaSeq platform.

Paired‐end Illumina reads were cleaned using the gDAT pipeline (Vasar *et al*., 2021). Raw reads were demultiplexed into samples using an 8‐bp barcode, allowing one nucleotide mismatch in both barcode pairs of the forward and reverse reads. Demultiplexed reads were then screened for the correct forward and reverse primers, allowing for one nucleotide mismatch in both pairs. Paired‐ended reads were retained if their average quality score was ≥ 30. Orphan reads, in which only one pair passed the filtering process, were discarded. Filtered paired‐end reads were merged using flash (v.1.2.11) (Magoč & Salzberg, 2011) with default parameters (overlap ≥ 10 bp, identity ≥ 75%). Chimeric sequences were identified and removed by checking them against the UNITE database (v.10.0; Abarenkov *et al*. (2024)) with vsearch (v.2.15.0; Rognes *et al*. (2016)). The ITS2 target marker region reads were clustered at 97% identity using VSEARCH, and cluster centroids were identified against the UNITE database (v.910.0) with an 80% alignment threshold. Taxonomic identification was assigned at the species (97% identity), genus (95% identity), and family (90% identity) levels. ITS2 target marker region operational taxonomic units (OTUs) were assigned to guilds using the FungalTraits database (Põlme *et al*., 2020). We use the term putative pathogens to describe fungal taxa assigned to the plant pathogen guild. This terminology acknowledges that guild assignments are based on literature, primarily from agricultural studies, and that the pathogenicity of these taxa within our study populations has not been empirically verified. The data were filtered to exclude OTUs with minimum abundance below 0.005%, and data were rarified to the minimum sequencing depth in the dataset (18 428 reads).

Raw reads from this Targeted Locus Study have been deposited in the National Center for Biotechnology Information Sequence Read Archive (BioProject PRJNA1244433).

### Statistical analyses

All statistical analyses were performed in R (R Core Team, 2024).

We fitted linear regression models with grassland age group as a fixed factor to assess differences in soil abiotic (soil texture, pH, total organic carbon, total nitrogen, ammonium‐N, nitrate‐N, Olsen‐phosphorus, and total phosphorus) and biotic (Shannon diversity and relative abundance of putative fungal pathogens and AM fungi) properties across the 15 sites, using the *lm* function in base R. We also performed permutational multivariate analysis of variance (PERMANOVA) to evaluate the effect of grassland age on fungal community composition using the *adonis* function from the vegan package (Oksanen *et al*., 2025). A Bray–Cutis distance matrix of relative abundance data for all fungi, putative pathogens, and AM fungi were computed using the *vegdist* function. We then performed Principal Coordinates Analysis (PCoA) on this distance matrix using the *cmdscale* function. To describe which OTUs correlated with the PCoA axes, we used the *envfit* function from the vegan package, testing OTUs that occurred in at least five soil samples.

We fitted linear mixed‐effects models to analyze the influence of experimental drought on CO_2_ fluxes (photosynthetic rate, ecosystem respiration, and NEE), shoot and root biomass, and mean plant population traits (leaf and root traits, and AM fungal root colonization) at the mesocosm level, using the *lmer* function from the lme4 package (Bates *et al*., 2015). Fixed effects included drought treatment, soil age group, plant–soil combination (‘home’ vs ‘away’), and their interactions. We included random intercepts for both soil and plant origin sites and allowed the effect of drought treatment to vary among sites by specifying drought treatment as a random slope (response ~ drought × soil age group × plant–soil combination + (drought|soil origin site) + (drought|plant origin site)). For photosynthetic rate and NEE, we also included the interaction between drought treatment and PAR (drought × PAR) as a fixed factor to account for variation in light availability. Parallel models were fitted, replacing soil age group with plant age group, to test for the effect of plant population age on measured parameters, thereby distinguishing potential evolutionary changes in plant populations from those driven by soil age, local plant–soil adaptation, or their interaction.

To identify the best microbial predictors underlying the soil age effect on CO_2_ fluxes and aboveground productivity, and their responses to drought, we selected soil microbial community properties that differed significantly across grassland age groups in soils used to set up the drought experiment. We used Akaike information criterion (AIC) to compare alternative linear mixed models, as described previously, replacing soil age group with each selected microbial variable.

We tested the trade‐off between growth potential under moist conditions and drought resilience across individual *B. media* genotypes. Genotype‐level drought resilience within a given plant–soil combination was calculated as the natural log‐transformed ratio of final shoot dry mass of each genotype in the drought treatment to that under control conditions (Van Ruijven & Berendse, 2010). The drought resilience was then regressed against shoot biomass and LDMC under moist conditions, reflecting fast and resource‐conservative growth strategies, respectively (Bristiel *et al*., 2018). When evaluating the relationship between trait responses to a treatment (in our case, drought) and trait values under control conditions, regression to the mean can lead to overestimation of treatment effects by misattributing random variation to biological responses (Kelly & Price, 2005). To correct for this bias and ensure more accurate estimates of drought resilience, we applied a regression‐to‐the‐mean adjustment using an R script by Gunderson (2023). The adjusted drought resilience was used as the response variable in a linear mixed‐effects model. Fixed factors included grassland soil age group (or plant population age group), plant–soil combination, and either shoot dry mass or LDMC under control conditions, along with their interactions. Random factors included soil site origin, plant genotype identity (nested within site origin), and mesocosm identity (adjusted drought resilience ~ shoot biomass (or LDMC) under control × soil (or plant population) age group × plant–soil combination + (1|soil origin site) + (1|genotype identity) + (1|mesocosm)).

For the linear mixed‐effects models, we removed the random effects if their variance was estimated as zero. We simplified models by removing nonsignificant fixed terms and random slopes based on AIC and the likelihood ratio test (*P* > 0.05; function *anova*). Fixed‐effect significance was assessed using the *Anova* function from car package (Fox *et al*., 2012), and group differences were tested with Tukey's *post hoc* comparisons using the *emmeans* function from emmeans package (Lenth & Lenth, 2018). Model fit was evaluated by calculating marginal *R* ^2^ (variance explained by fixed effects) and conditional *R* ^2^ (variance explained by the full model) using the *performance* function from performance package (Lüdecke *et al*., 2021). Model assumptions were checked by visual inspection of residual vs fitted plots and Q‐Q plots to assess homoscedasticity and normality of residuals.

## Results

### Soil properties and microbial communities across grasslands of different age

**Fig. 2:**
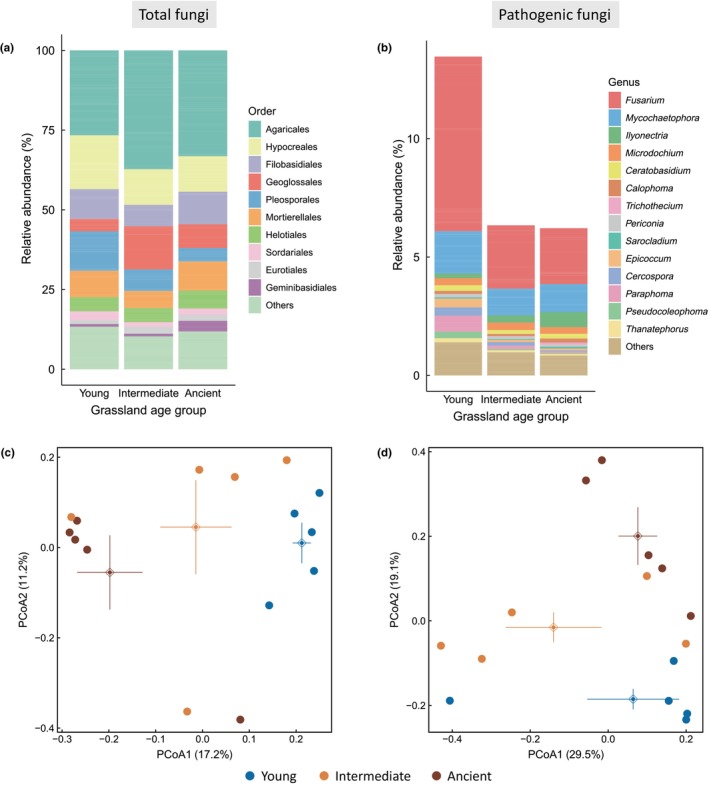
Soil fungal communities across the three grassland age groups. (a) illustrates the relative abundance of the 10 dominant fungal orders and the pooled abundance of remaining orders (‘Others’) across grassland age groups. (b) displays the relative abundance of the 10 most abundant pathogenic fungal genera and the combined abundance of remaining genera (‘Others’) within each grassland age group. For both panels, the relative abundance values represent means calculated from five replicate sites of each grassland age group. Principal Coordinates Analysis (PCoA) plots depict variation in total (c) and pathogenic fungal (d) community composition across three grassland age groups. Variation in fungal community composition explained by the first and second PCoA axes is shown in brackets. Dots denote individual observations, and dots with bidirectional error bars show mean values ± SE for the first and second principal coordinates for each grassland age group.

The three grassland age groups (young, intermediate, and ancient) did not show a significant difference in soil texture and nutrient content (Table S1). All soils had approximately neutral pH with young grassland soils exhibiting significantly higher pH than intermediate and ancient grassland soils (*F*_2,12_ = 6.76, *P* = 0.011; Table S1). The diversity of soil fungal communities and putative pathogens was comparable among the three age groups (Fig. 2a,b; Table S2). However, young grassland soils hosted a significantly higher relative abundance of putative fungal pathogens than intermediate and ancient grassland soils (on average 13.5, 6.3, and 6.2% for young, intermediate, and ancient soils, respectively; *F*_2,12_ = 8.26, *P* = 0.006; Fig. 2b; Table S2). Young grasslands were also characterized by higher relative abundance and diversity of AM fungi (*F*_2,12_ = 6.27, *P* = 0.014, Fig. S2a; and *F*_2,12_ = 6.26, *P* = 0.014, Table S2; respectively); however, the overall abundance of AM fungal sequences was mostly low (median 0.2%, range 0.02–1.2%). PERMANOVA showed that grassland age significantly influenced the community composition of all fungi, putative pathogens, and AM fungi (*F*_2,12_ = 1.61, *P* = 0.003; *F*_2,12_ = 1.97, *P* = 0.023; *F*_2,12_ = 1.44, *P* = 0.004; respectively; Figs 2c,d, S2b).

### Response of CO_2_ fluxes to drought and its relationship with soil microbial communities

**Fig. 3:**
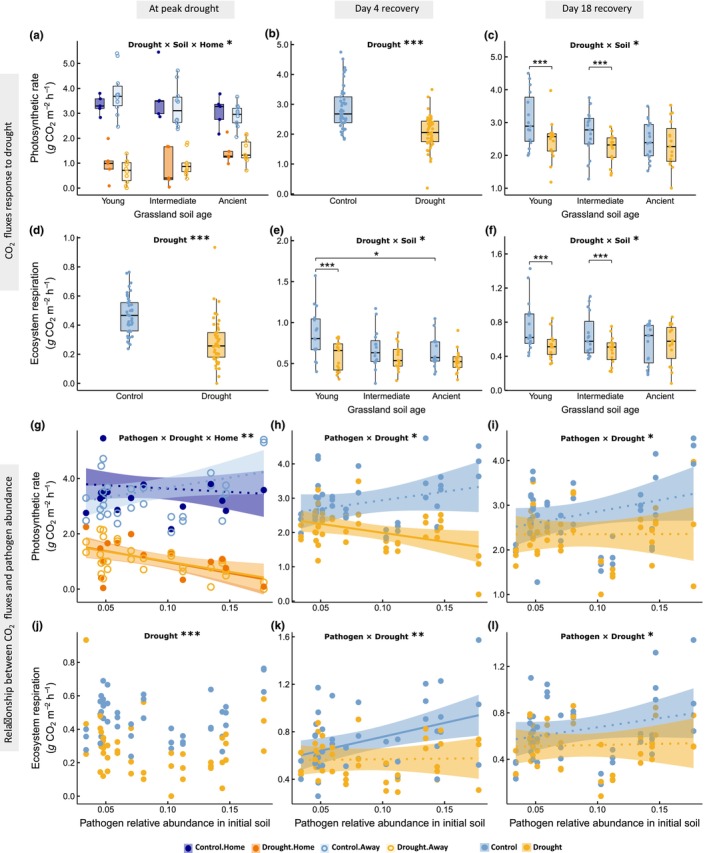
Response of CO_2_ fluxes to drought and its dependence on grassland soil age group, plant–soil combination, and the relative abundance of putative fungal pathogens in the initial grassland soils. Photosynthetic rate and ecosystem respiration of the mesocosms at the peak drought, on the 4th and the 18th day of recovery from drought are shown. Figure displays variables with significant effects in the model. The dots represent individual observations; blue and yellow indicate mesocosms under control and drought conditions, respectively. For significant three‐way interactions, filled symbols and darker colors represent plant populations of *Briza media* grown in ‘home’ soils from their site of origin, while open symbols and lighter colors indicate those in ‘away’ soils from other age groups. (a–f) The box plot shows the median value and first and third quartiles of the data. The whisker lines extend to 1.5 times the interquartile range, excluding outliers. (g–l) The solid line indicates the regression line with significant slope (*P* < 0.05) and the dotted line shows the regression line with non‐significant slope (*P* > 0.05). The shaded area displays 95% confidence interval of the fitted line. Significant model parameters and *post hoc* test results are presented with significance level indicators: *, *P* < 0.05; **, *P* < 0.01; ***, *P* < 0.001.

At peak drought, photosynthetic rate and NEE were significantly affected by the interaction among drought treatment, grassland soil age group, and plant–soil combinations (*F*_2,43_ = 4.59, *P* = 0.016 and *F*_2,43_ = 4.52, *P* = 0.016, respectively; Figs 3a, S3a). The negative effect of drought on photosynthetic rate and NEE was weaker in plants grown in soils from ancient grasslands. In young grassland soils, the decline in photosynthesis and NEE was less pronounced in ‘home’ than in ‘away’ plant–soil combinations (Figs 3a, S3a). Ecosystem respiration was reduced under drought, regardless of grassland age and plant–soil combinations (*F*_1,64_ = 49.65, *P* < 0.001; Fig. 3d). On day 4 of recovery, the negative post‐drought effect on ecosystem respiration was significant only in mesocosms with young grassland soil (Fig. 3e). After 18 d of recovery, photosynthetic rate and ecosystem respiration had fully recovered in mesocosms with soil from ancient, but not from young and intermediate grasslands (*F*_2,61_ = 4.77, *P* = 0.012, and *F*_2,62_ = 3.35, *P* = 0.041, respectively; Fig. 3c,f). Plant population age did not significantly affect CO_2_ fluxes, except for photosynthesis and respiration at peak drought, where populations from the intermediate age group tended to show the strongest drought response (*F*_2,41_ = 4.60, *P* = 0.016, and *F*_2,57_ = 3.46, *P* = 0.038, respectively; Table S3; Fig. S4).

The response of CO_2_ fluxes to drought was best predicted by the relative abundance and composition of putative pathogen communities in the initial grassland soils (Table S4; Figs 3, S3 and S5). At peak drought, photosynthetic rate declined more strongly in soils with higher relative abundance of putative pathogens, particularly in ‘away’ plant–soil combinations (three‐way interactive effect between drought, pathogen abundance, and plant–soil combination, *F*_1,45_ = 8.66, *P* = 0.005; Fig. 3g). A similar relationship was observed for NEE (*F*_1,46_ = 8.17, *P* = 0.006; Fig. S3d). On days 4 and 18 of recovery, the negative post‐drought effect on photosynthesis and respiration was more pronounced in soils with higher pathogen abundance (Day 4: *F*_1,13_ = 7.49, *P* = 0.017, and *F*_1,63_ = 10.13, *P* = 0.002, respectively; Day 18: *F*_1,62_ = 6.94, *P* = 0.011, and *F*_1,62_ = 4.52, *P* = 0.038, respectively; Fig. 3h,i,k,l).

### Response of plant productivity to drought and its relationship with soil microbial communities

**Fig. 4:**
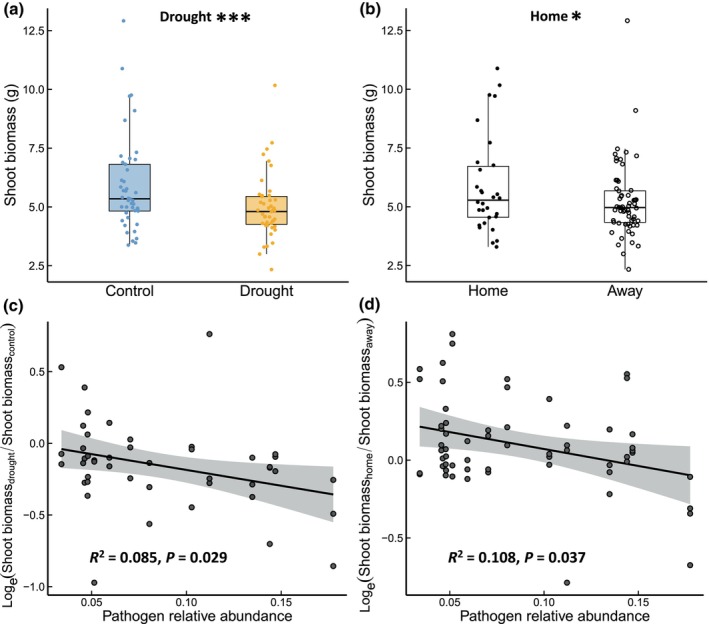
Responses of aboveground productivity to drought (a) and to growing in ‘home’ vs ‘away’ soil (b), and the relationship between these responses and the relative abundance of putative fungal pathogens in initial grassland soils (c, d). The dots denote individual observations. The box plot shows the median value and first and third quartiles of the data. The whisker lines represent the data range within 1.5 times the interquartile range, excluding outliers. Significant model parameters are shown with significance level indicators: *, *P* < 0.05; ***; *P* < 0.001. Aboveground productivity response to soil origin was calculated as the natural log‐transformed ratio of shoot biomass of a given *Briza media* population when grown in ‘home’ soil from the same site to that in ‘away’ soil from other age groups. Aboveground productivity response to drought was calculated as the natural log‐transformed ratio of shoot biomass of a given plant population in the drought treatment to that under non‐limiting water control conditions. The solid line indicates the regression line, and shaded area displays 95% confidence interval of the fitted line. *R* ^2^ accounts for the proportion of variance explained by fixed effects.

At the end of the experiment, aboveground productivity was, on average, 16% lower in drought‐treated mesocosms (*F*_1,73_ = 13.49, *P* < 0.001; Fig. 4a). In addition, aboveground productivity was, on average, 12% higher in plants grown in ‘home’ soils from their site of origin than in ‘away’ soils from other age groups, regardless of drought treatment or grassland age (*F*_1,73_ = 4.93, *P* = 0.030; Fig. 4b; Table S5). The home soil advantage was more pronounced at low relative abundance of soil fungal pathogens (*F*_1,13_ = 5.39, *P* = 0.037, *R* ^2^ = 0.108; Fig. 4d). The response of aboveground productivity to drought was best predicted by the relative abundance and composition of putative pathogen communities in the initial grassland soils (Table S6). Specifically, drought resilience for aboveground productivity was higher when plants were grown in soil with lower relative pathogen abundance (*F*_1,43_ = 5.08, *P* = 0.029, *R* ^2^ = 0.085; Fig. 4c) and was also significantly associated with putative pathogen communities along the second PCoA axis (*F*_1,43_ = 5.09, *P* = 0.029, *R* ^2^ = 0.085; Fig. S6). We identified 34 OTUs from 18 genera that significantly correlated with the second PCoA axis (Table S7). Of these, 21 OTUs negatively correlated with this axis, declining in abundance with grassland age. Twelve OTUs belonged to genus *Fusarium*, with 11 declining and one increasing along this axis (Table S7). Additionally, the relative abundance of eight OTUs correlated significantly with drought resilience of plant aboveground productivity (Table S7).

### Response of plant traits and belowground biomass to drought

**Fig. 5:**
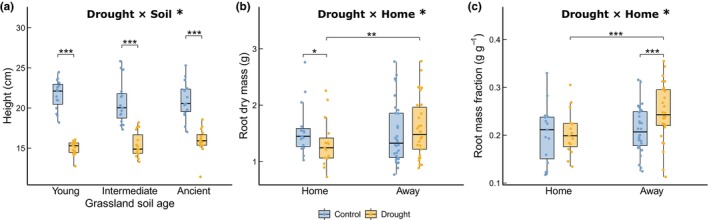
Plant traits are modified by drought, grassland soil age, and plant–soil combination (plants grown in ‘home’ soil from their site of origin or ‘away’ from other age groups). Figure displays variables with significant effects in the model. The dots denote individual observations. The box plot shows the median value and first and third quartiles of the data. The whisker lines display the range of data within 1.5 times the interquartile range, excluding outliers. Significant model parameters and *post hoc* test results are shown with significance level indicators: *, *P* < 0.05; **, *P* < 0.01; ***, *P* < 0.001.

The impact of drought on mean plant height varied with grassland soil age, with a greater decline in plants grown in young grassland soils compared with older grassland soils (*F*_2,62_ = 3.73, *P* = 0.030; Fig. 5a). Leaf nitrogen content and RTD increased significantly in the drought treatment but were not affected by grassland age (Fig. S7a,b). Drought significantly reduced root dry mass in plants grown in their ‘home’ soils, whereas plants in ‘away’ soils had similar root mass under control and drought treatments (*F*_1,72_ = 6.41, *P* = 0.014; Fig. 5b). Allocation to root mass did not differ between control and drought treatments in populations grown in ‘home’ soils, but increased under drought in plants grown in ‘away’ soils (*F*_1,72_ = 4.34, *P* = 0.041; Fig. 5c). Root diameter was lower in plants grown in ‘home’ than in ‘away’ soils (*F*_1,74_ = 16.83, *P* < 0.001; Fig. S7c). AM fungal root colonization was not significantly affected by drought, plant–soil combination, or grassland age, except for a significantly lower vesicle abundance in young grassland soils (*F*_2,12_ = 5.51, *P* = 0.020). Plant population age did not significantly affect biomass production or any other traits, except for SRL, in which drought significantly reduced SRL only in plants from intermediate age groups (Fig. S7d; Table S5).

### Genotype‐level drought resilience and its relationship with plant traits under nonlimiting water conditions

**Fig. 6:**
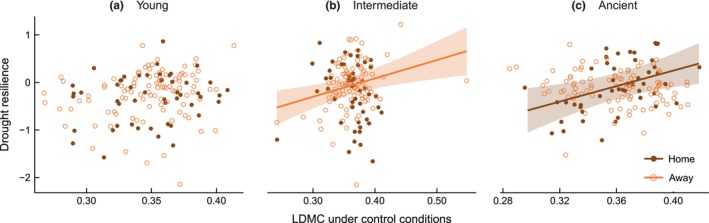
Relationship between drought resilience and leaf dry matter content under nonlimiting water conditions across genotypes of *Briza media* grown in soils originating from (a) young, (b) intermediate, and (c) ancient grasslands. Drought resilience was calculated for each genotype as the natural log‐transformed ratio of shoot dry mass in the drought treatment to that under control conditions. Dots represent individual observations. Filled brown symbols represent plant genotypes grown in ‘home’ soils from their site of origin, whereas open orange symbols indicate genotypes grown in ‘away’ soils from other age groups. Solid lines show significant relationships, and shaded areas indicate 95% confidence interval of the fitted lines.

Genotype‐level drought resilience was not significantly related to grassland soil age group (*F*_2,38_ = 1.32, *P* = 0.279), plant population age group (*F*_2,42_ = 0.07, *P* = 0.934), or ‘home’ vs ‘away’ plant–soil combination (*F*_1,37_ = 0.45, *P* = 0.508). Shoot biomass under nonlimiting water conditions did not differ significantly among grassland soil (*F*_2,36_ = 2.25, *P* = 0.120) and plant population (*F*_2,45_ = 0.202, *P* = 0.818) age groups, or plant–soil combinations (*P* > 0.5). LDMC under nonlimiting water conditions tended to be lower for populations grown in young soils (*F*_2,35_ = 3.282, *P* = 0.049) but did not differ significantly among plant population age groups (*F*_2,50_ = 0.533, *P* = 0.590) or plant–soil combinations (*P* > 0.5). Across all genotypes, drought resilience was negatively related to shoot biomass under nonlimiting water conditions (*F*_1,437_ = 7.21, *P* = 0.008, *R* ^2^ = 0.016; Fig. S8). However, the relationship between drought resilience and LDMC under nonlimiting water conditions depended on grassland soil age group and plant–soil combination (*F*_2,421_ = 5.43, *R* ^2^ = 0.072, *P* = 0.005; Fig. 6). A significant positive relationship was only found for plant populations from ancient grasslands in their ‘home’ soils (*R* ^2^ = 0.120, *P* = 0.018; Fig. 6c), and for ‘away’ plant–soil combinations in intermediate grassland soils (*R* ^2^ = 0.065, *P* = 0.004; Fig. 6b).

## Discussion

In agreement with our predictions, mesocosms containing ancient grassland plant populations and soils were more resistant to drought, as evidenced by their ability to better sustain photosynthesis and carbon fluxes during drought and recovered faster after drought. Contrary to our predictions, we found no evidence of an evolutionary shift toward more resource‐conservative traits or higher drought resilience in plant populations as grassland age increased. Instead, enhanced drought resilience was mainly driven by soil conditions. Specifically, the greater drought resilience of CO_2_ fluxes in ancient grassland soils was explained by the lower relative abundances of putative soil fungal pathogens compared with younger soils. Additionally, the composition of putative pathogen communities shifted with grassland age, contributing to changes in drought response. A previous study demonstrated a reduction in the relative abundances of soil fungal pathogens in grasslands that had ceased arable cultivation 7–30 years ago (Hannula *et al*., 2017). Our study shows that successional changes in fungal communities continue at a longer timescale, potentially taking centuries to reach the state of ancient grasslands. These changes could be a key process contributing to reduced drought resilience in successional compared to ancient grasslands. Moreover, responses of CO_2_ fluxes to drought were significantly influenced by whether plants were grown in their ‘home’ or ‘away’ soil, indicating the important role of local co‐adaptation between plants and soil microbial communities in shaping drought resilience of grassland populations.

Pathogens are known to negatively affect plant growth (Borowicz, 2001) and photosynthesis (Yang & Luo, 2021). In the present study, however, the negative impact of high pathogen abundance on photosynthetic rate and net ecosystem carbon flux was significant only under drought conditions, not under well‐watered control conditions. This finding supports a growing body of evidence, particularly from agricultural systems, showing that the combined stress of drought and pathogens exerts a stronger negative effect on plant growth than would be expected from their individual impacts (de Vries *et al*., 2023; Choudhary & Senthil‐Kumar, 2024). This differential response may result not only from plant physiological response to drought but also from drought‐induced reduction in carbon allocation to beneficial microorganisms such as AM fungi, which are known to protect against pathogens (Karlowsky *et al*., 2018). Under well‐watered conditions, beneficial microorganisms may protect plants effectively against soil‐borne pathogens (Newsham *et al*., 1995; Cameron *et al*., 2013; Pieterse *et al*., 2014) and mitigate their negative impacts (Liu *et al*., 2021). By contrast, during drought, AM fungi may be more adversely affected than pathogenic fungi (Ochoa‐Hueso *et al*., 2018; Lozano *et al*., 2021), potentially weakening their protective role. While several studies have shown that AM fungi can alleviate drought stress in plants (Püschel *et al*., 2020; Tang *et al*., 2023), AM fungal colonization remained unchanged under drought in our study, suggesting no detectable AM fungi‐mediated drought responses in this system. However, both root colonization assays and ITS sequencing have limitations in capturing AM fungal function, and more sensitive or functionally targeted approaches (e.g. isotopic tracing) may be required to detect the potential AM fungal benefits in our system. In addition, other microbial taxa, such as beneficial and pathogenic bacteria, may have contributed to the modulation of drought responses and deserve exploration in future studies (Qi *et al*., 2022; Xiang *et al*., 2025).

Local co‐adaptation or reassembly between plant populations and microbial communities also contributed to plant productivity and drought responses of ecosystem functions across the grassland age groups. We found that plants generally showed higher aboveground productivity when grown in their ‘home’ soils, reflecting a performance advantage that depends on the matching between plant population and soil origin and demonstrating a classic example of local adaptation to soil conditions (Rúa *et al*., 2016). The absence of significant interactions between grassland age and plant–soil combinations indicates that this local adaptation was evident across all grassland ages. The home soil advantage was more pronounced in soils with lower pathogen abundance (Fig. 4d), indicating that soil pathogens may be an important selective agent in this study system. We also found that drought caused a milder reduction in carbon fluxes in young soils with high pathogen abundance when paired with ‘home’ compared with ‘away’ plant populations (Fig. 3g). Such home advantage for young grassland plant populations suggests that these populations may have evolved enhanced resistance to local pathogens (Mursinoff & Tack, 2017), whereas older populations, typically exposed to lower pathogen pressure, may be less adapted to such stress.

Plant strategies for coping with drought were also modulated by plant–soil local adaptation, regardless of grassland age. We found that plants exhibited similar drought resilience for biomass production in both ‘home’ and ‘away’ soils, but achieved it through different mechanisms. Specifically, despite an overall reduction in growth under drought conditions, plants in ‘away’ soil increased biomass allocation to roots (Fig. 5c), suggesting an adaptive response to drought stress (Comas *et al*., 2013). By contrast, plants grown in their ‘home’ soils showed no significant change in biomass allocation to roots under drought, indicating that local adaptation between plant genotypes and their native soil conditions may have alleviated water stress.

Contrary to our expectation, we did not observe significant shifts toward more conservative, drought‐tolerant traits with increasing grassland age. Nevertheless, LDMC, a trait associated with leaf water conservation and desiccation resistance (Markesteijn *et al*., 2011), was a significant predictor of drought resilience in specific plant–soil combinations. Genotypes with higher LDMC were more drought resilient, but this relationship was context‐dependent: the positive correlation between LDMC and drought resilience was most pronounced when genotypes from ancient grasslands were grown in their ‘home’ soils (Fig. 6c). This correlation aligns with interspecific findings showing that plant species with high LDMC exhibit enhanced drought resilience (Blumenthal *et al*., 2020; Májeková *et al*., 2021; Mount *et al*., 2024). Our results suggest that the association between LDMC and drought resilience is additionally influenced by local plant–soil interactions. In ancient grasslands, long‐term co‐adaptation between plant and soil biota may have strengthened the positive relationship between LDMC and drought resilience, as coevolved soil organisms may enhance the functional benefits of conservative leaf traits under stress. By contrast, recently assembled soil communities in younger grasslands may provide weaker support under drought, resulting in a weakened link between LDMC and drought resilience. The role of soil biota in mediating plant adaptation to drought conditions has been detected in other studies, although it has not yet been linked to leaf traits such as LDMC (Gehring *et al*., 2017; Remke *et al*., 2021).

Overall, our findings suggest that soil conditions, particularly changes in the relative abundance and composition of soil fungal pathogens with grassland age, can play a more significant role than intraspecific plant trait variation in determining the resilience of plant population productivity and carbon fluxes to drought. In addition, local adaptation between plant populations and soils was detectable across all grassland age groups and influenced plant productivity and plant drought responses by modulating plant–microbial interactions and resource allocation strategies. Previous studies have highlighted the ecological significance of ancient grasslands and potential tipping points in grassland degradation that can make restoration challenging (Buisson *et al*., 2022). Here, we provide additional evidence of the value of ancient grasslands during climate change by demonstrating their superior resilience to drought. However, we also identify a challenge for grassland restoration: the increased drought resilience of plant populations in ancient grasslands is associated with lower soil pathogen abundance. This finding underscores the importance of considering long‐term microbial succession alongside the well‐recognized role of plant functional traits and species richness in restoring climate‐resilient grasslands (Fry *et al*., 2018; Lüscher *et al*., 2022; Oram *et al*., 2023). Future research should explore the mechanisms by which microbial communities influence grassland drought resilience and investigate how drought alters soil microbial communities.

## Competing interests

None declared.

## Author contributions

YJ, KK, HCP, and MS designed the research. YJ, JLE, PK, LL, MO, LP, AT, MT, JW, NX, KK, HCP, and MS collected the data. MV contributed to the analysis of the sequencing data. YJ and MS wrote the first draft. All authors contributed to reviewing and editing the manuscript.

## Disclaimer

The New Phytologist Foundation remains neutral with regard to jurisdictional claims in maps and in any institutional affiliations.

## Supporting information


**Fig. S1** Senescence of *Briza media* leaves at the peak of drought.
**Fig. S2** Mean relative abundance of AM fungal taxa and Principal Coordinates Analysis (PCoA) of AM fungal community detected in three age groups of grassland soil.
**Fig. S3** Response of CO_2_ fluxes to drought and its dependence on grassland soil age group and plant–soil combination, and the relative abundance of putative fungal pathogens in the initial grassland soils.
**Fig. S4** CO_2_ fluxes at peak drought across plant population age groups.
**Fig. S5** Relationship between drought response of mesocosm CO_2_ fluxes and the second principal coordinate of pathogen community composition in the initial grassland soils.
**Fig. S6** Relationship between drought resilience for mesocosm populations and the second principal coordinate of pathogen community composition in the initial grassland soils.
**Fig. S7** Response of plant traits to drought and its dependence on the plant population age group and plant–soil combinations.
**Fig. S8** Relationship between drought resilience and shoot biomass under non‐limiting water conditions across genotypes of *Briza media*.
**Methods S1** Characterization of field‐collected soil.
**Methods S2** Measurement of plant traits.
**Table S1** Soil abiotic properties of the grasslands.
**Table S2** Soil fungal communities in the grassland soils.
**Table S3** Summary of linear mixed effects models analyzing the effects of drought, plant–soil combination, plant population age group and their interactions on CO_2_ fluxes.
**Table S4** Selection of the best predictors of drought response of CO_2_ fluxes.
**Table S5** Summary of linear mixed effects models analyzing the main effects of drought, plant–soil combination, plant population age group and their interactions on plant traits and AM fungal colonization in roots.
**Table S6** Selection of the best predictors of drought response of aboveground productivity.
**Table S7** The list of operational taxonomic units (OTUs) that significantly correlated with the second axis of Principal Coordinates Analysis (PCoA) of putative fungal pathogen composition in 15 soils used in the study, and the correlation between the relative abundance of the OTUs and plant drought resilience.
Please note: Wiley is not responsible for the content or functionality of any Supporting Information supplied by the authors. Any queries (other than missing material) should be directed to the *New Phytologist* Central Office.

## Data Availability

The data and code supporting the results are archived in Figshare and are available at: https://doi.org/10.6084/m9.figshare.32043174. Raw sequencing reads from this Targeted Locus Study have been deposited in the National Center for Biotechnology Information Sequence Read Archive (BioProject PRJNA1244433).
