# Supplementary material for: Grassland age and local adaptation shape drought resilience across semi‐natural grassland populations
Source: New Phytol. 2026 Apr 22;250(6):3646–60. doi: 10.1111/nph.71196 (PMC13193343; doi:10.1111/nph.71196)
Supplement: Supplementary file 1 — Fig. S1 Senescence of Briza media leaves at the peak of drought. Fig. S2 Mean relative abundance of AM fungal taxa and Principal Coordinates Analysis (PCoA) of AM fungal community detected in three age groups of grassland soil. Fig. S3 Response of CO2 fluxes to drought and its dependence on grassland soil age group and plant–soil combination, and the relative abundance of putative fungal pathogens in the initial grassland soils. Fig. S4 CO2 fluxes at peak drought across plant population age groups. Fig. S5 Relationship between drought response of mesocosm CO2 fluxes and the second principal coordinate of pathogen community composition in the initial grassland soils. Fig. S6 Relationship between drought resilience for mesocosm populations and the second principal coordinate of pathogen community composition in the initial grassland soils. Fig. S7 Response of plant traits to drought and its dependence on the plant population age group and plant–soil combinations. Fig. S8 Relationship between drought resilience and shoot biomass under non‐limiting water conditions across genotypes of Briza media. Methods S1 Characterization of field‐collected soil. Methods S2 Measurement of plant traits. Table S1 Soil abiotic properties of the grasslands. Table S2 Soil fungal communities in the grassland soils. Table S3 Summary of linear mixed effects models analyzing the effects of drought, plant–soil combination, plant population age group and their interactions on CO2 fluxes. Table S4 Selection of the best predictors of drought response of CO2 fluxes. Table S5 Summary of linear mixed effects models analyzing the main effects of drought, plant–soil combination, plant population age group and their interactions on plant traits and AM fungal colonization in roots. Table S6 Selection of the best predictors of drought response of aboveground productivity. Table S7 The list of operational taxonomic units (OTUs) that significantly correlated with the second axis of Principal Coordinates An [file NPH-250-3646-s001.pdf]

New Phytologist Supporting Information

Article title: Grassland age and local adaptation shape drought resilience across semi-natural grassland populations

Authors: Yuying Jing, Jenalle L. Eck, Piia Kängsep, Lauri Laaspere, Miina Oras, Laura Puura, Anastasia Tõnisson, Mari Torsus, Martti Vasar, Jianlu Wu, Nianxun Xi, Kadri Koorem, Honor C. Prentice, Marina Semchenko

Article acceptance date: 29 March 2026

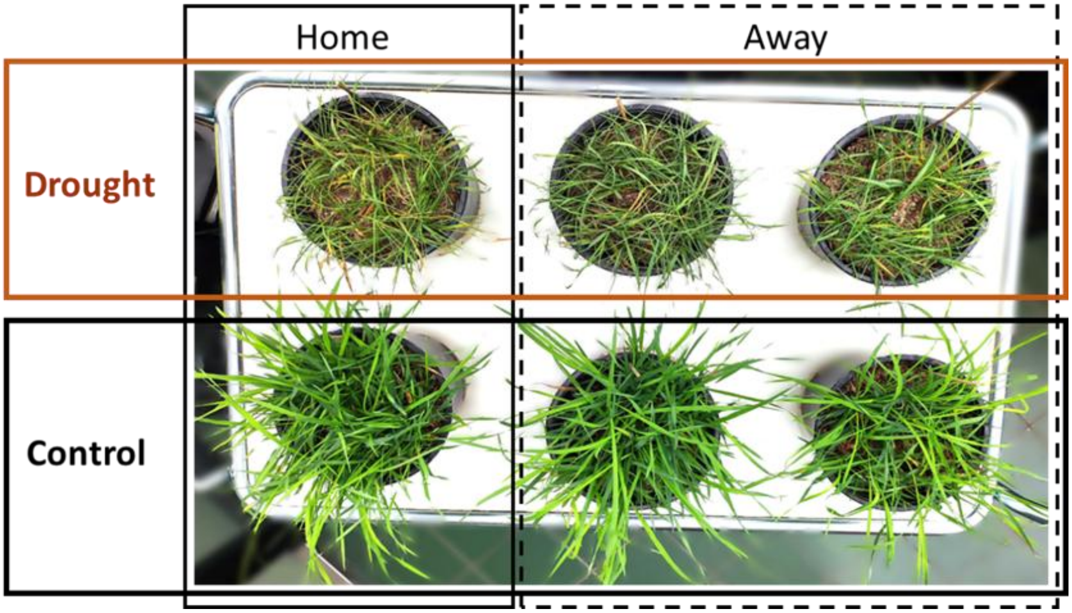

Figure S1 Senescence of *Briza media* leaves at the peak of drought. Plants under drought conditions (after 22 days of drought treatment; above: in "home" and "away" soil) are compared with those under non-limiting water control conditions (below: in "home" and "away" soil).

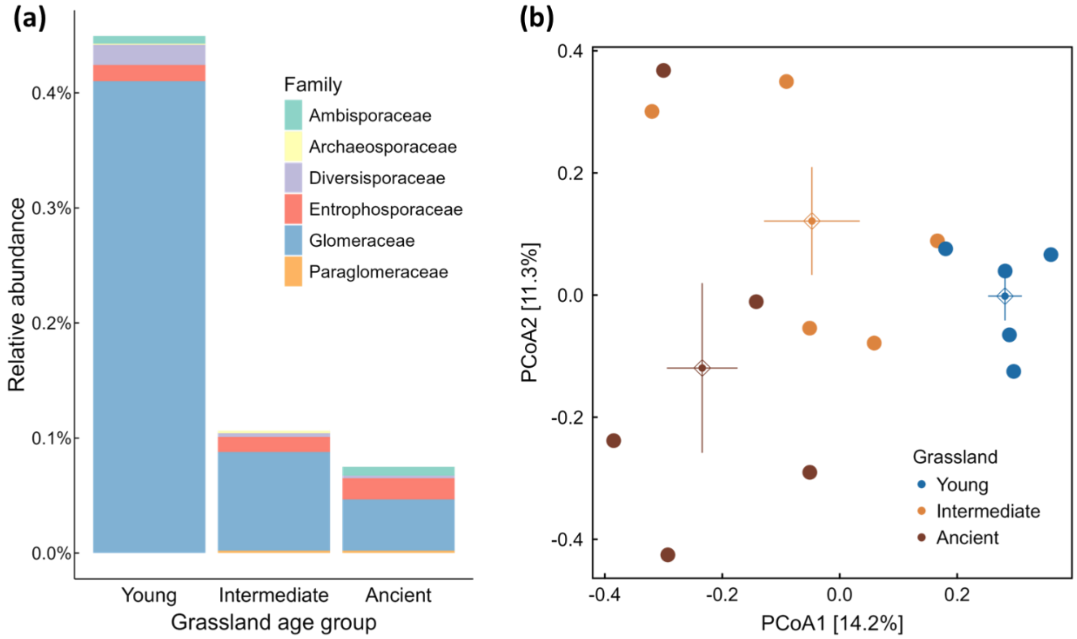

Figure S2 Mean relative abundance of AM fungal taxa (a) and Principal Coordinates Analysis (PCoA) of AM fungal community (b) detected in three age groups of grassland soil. Panel a displays the relative abundance of the ten most abundant AM fungal genera and the combined abundance of remaining genera ("Others") within each grassland age group. In panel b, dots with bidirectional error bars show mean values ± standard errors for PCoA1 and PCoA2 axes for each grassland age group. Variation in AM fungal community composition explained by the first and second PCoA axes is shown in brackets.

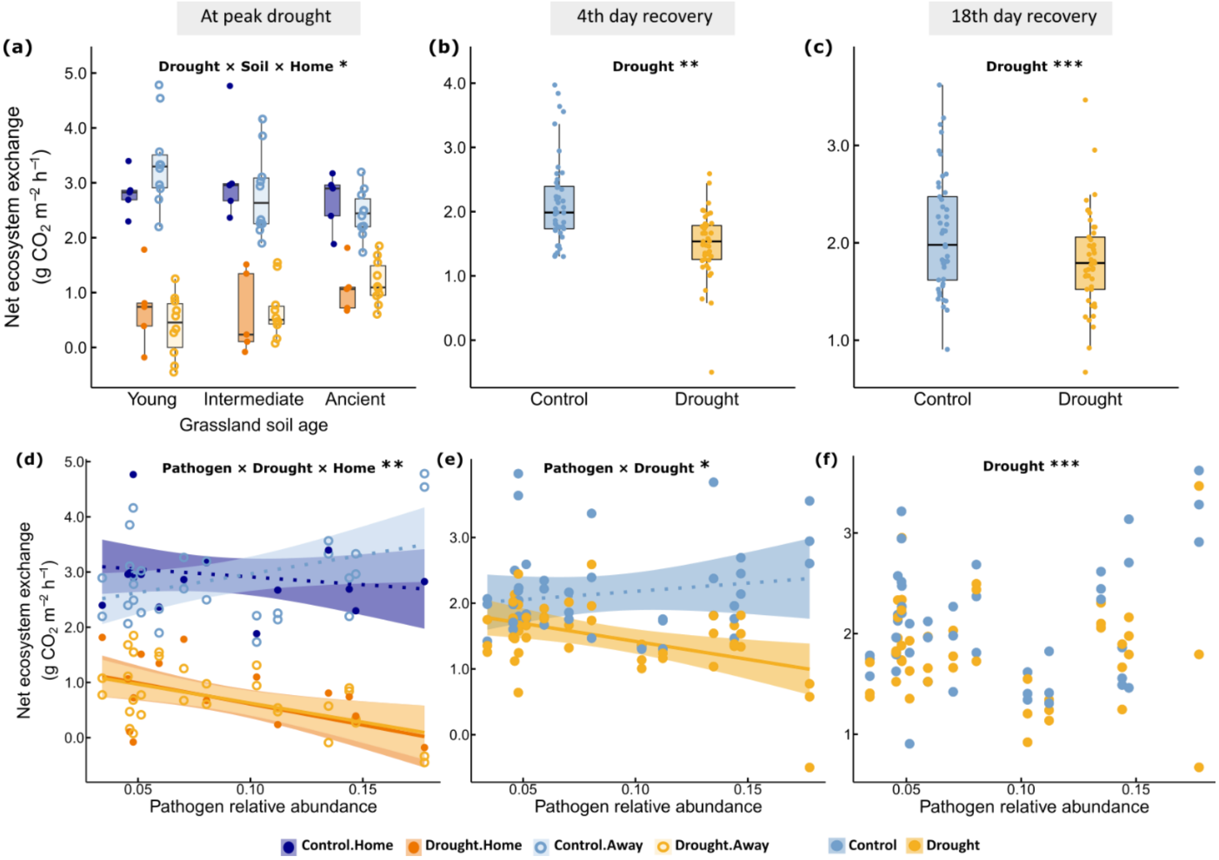

Figure S3 Response of CO_2 fluxes to drought and its dependence on grassland soil age group and plant-soil combination, and the relative abundance of putative fungal pathogens in the initial grassland soils. Net ecosystem exchange of the mesocosms at the peak drought, on the 4th day and the 18th day of recovery from drought are shown. The dots represent individual observations with blue and yellow colours indicating mesocosms subjected to control and drought conditions, respectively. In the case of significant three-way interaction, filled and open symbols, and darker and lighter shades of blue and orange indicate plant populations grown in "home" and "away" soils, respectively. (a–c) The box plot shows the median value and the first and third quartiles of the data. The whisker lines denote data range excluding outliers which extend beyond 1.5 times the interquartile range. (d–f) The solid line indicates the regression line with significant slope ($P < 0.05$) and the dotted line shows the regression line with non-significant slope ($P > 0.05$). The shaded area displays 95% confidence interval of the fitted line. Significant model parameters are presented in the figure with significant level indicators: $P < 0.05$, *; $P < 0.01$, **; $P < 0.001$, ***.

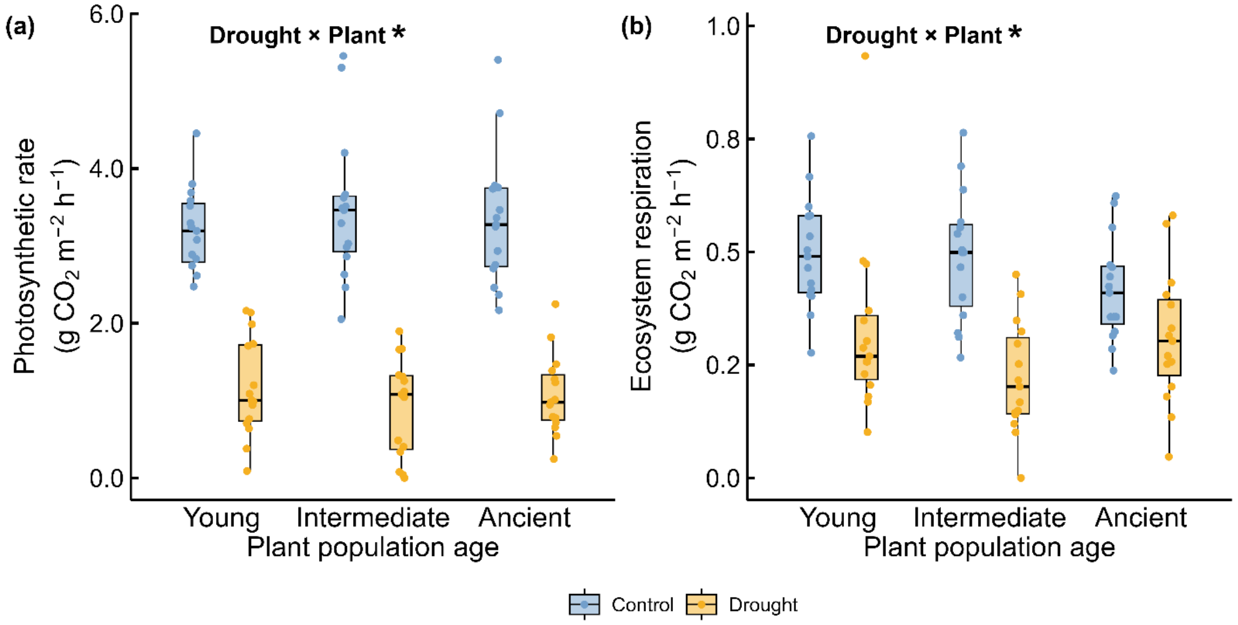

Figure S4 CO_2 fluxes at peak drought across plant population age groups. Photosynthetic rate (a) and ecosystem respiration (b) of the mesocosms at the peak drought are shown. The dots represent individual observations with blue and yellow colours indicating mesocosms subjected to control and drought conditions, respectively. The box plot shows the median value and the first and third quartiles of the data. The whisker lines denote data range excluding outliers which extend beyond 1.5 times the interquartile range. Significant model parameters are presented in the figure with significant level indicators: $P < 0.05$, *.

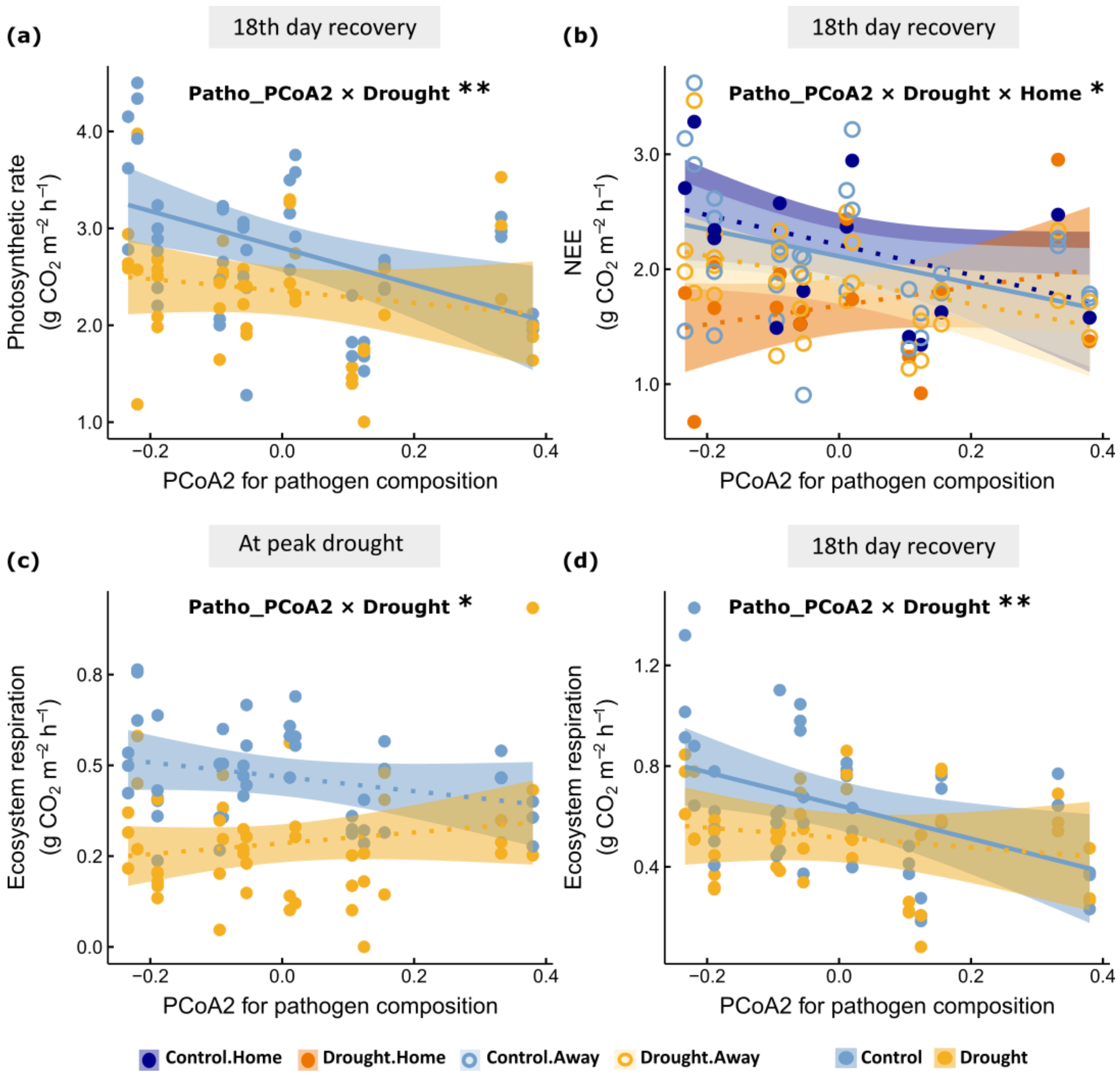

Figure S5 Relationship between drought response of mesocosm CO_2 fluxes and the second principal coordinate of pathogen community composition in the initial grassland soils. The solid line shows the regression line with significant slope ($P < 0.05$), and the dotted line shows the regression line with non-significant slope ($P > 0.05$). The shaded area displays 95% confidence interval of the fitted line. Significant model parameters are presented with significance level indicators: $P < 0.05$, *; $P < 0.01$, **. Patho_PCoA2, second principal coordinate of pathogen community composition. NEE, net ecosystem exchange.

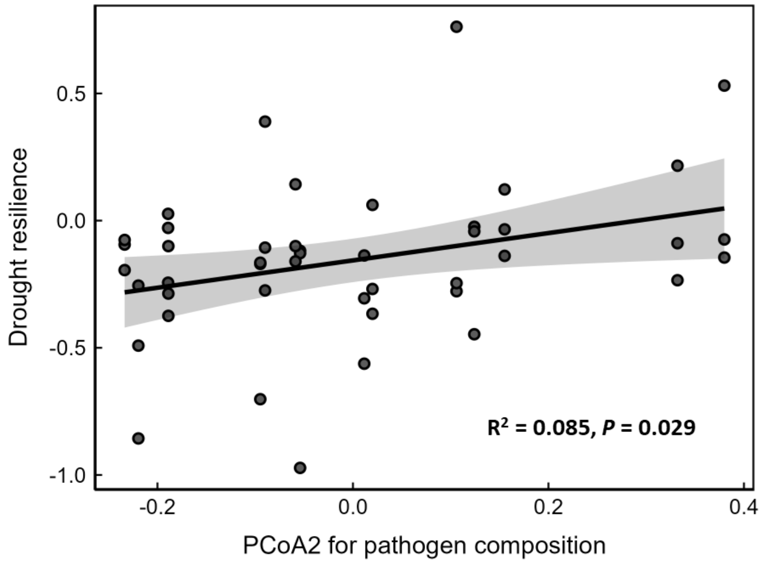

Figure S6 Relationship between drought resilience for mesocosm populations and the second principal coordinate of pathogen community composition in the initial grassland soils. Drought resilience was calculated as the natural log transformed ratio of aboveground biomass for each mesocosm in the drought treatment to that under control conditions. The dots denote individual observations. The solid line indicates the regression line, and shaded area displays 95% confidence interval of the fitted line. R^2 accounts for the proportion of variance explained by fixed effects.

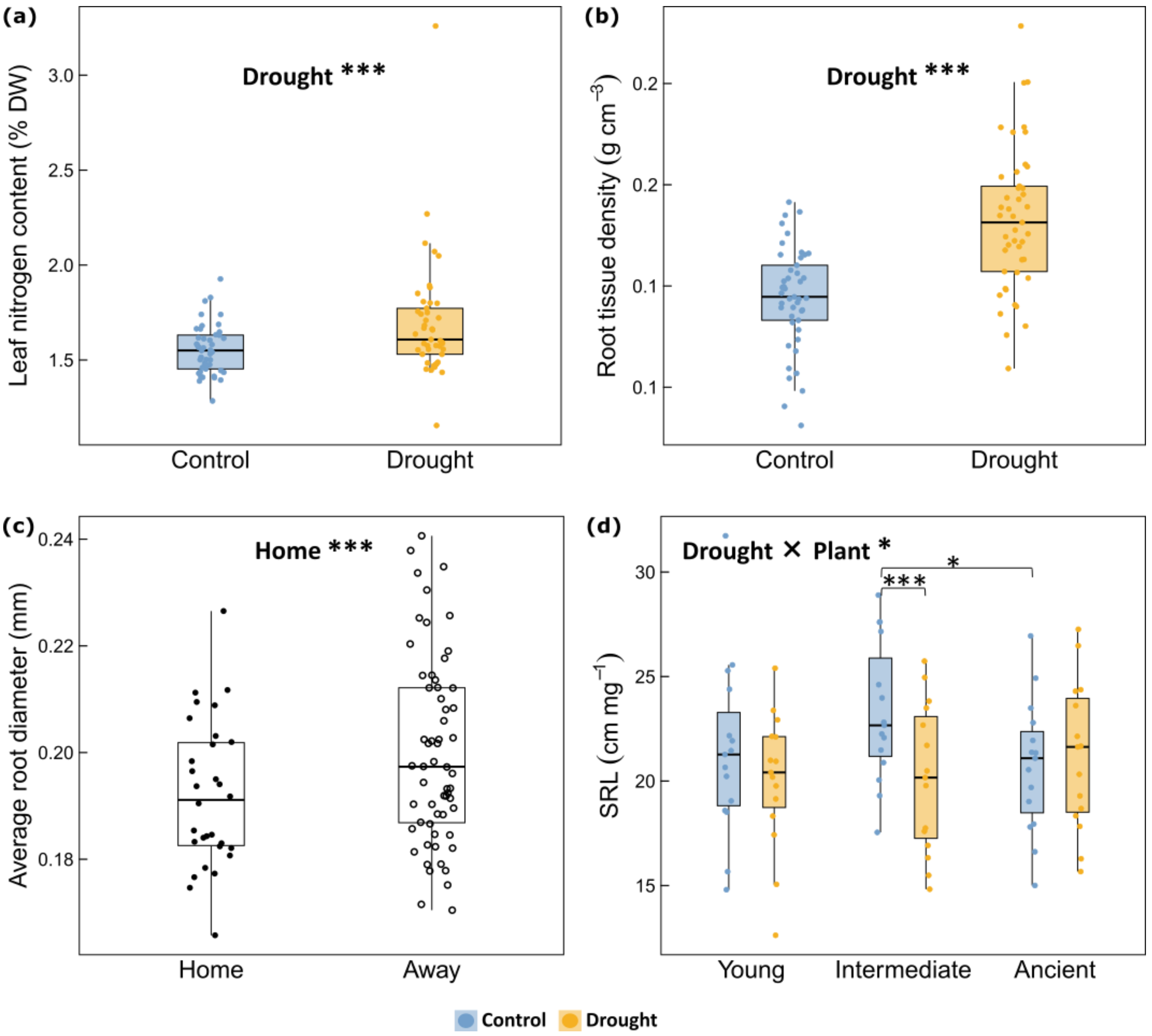

Figure S7 Response of plant traits to drought and its dependence on the plant population age group (*Briza media* genotypes originating from young, intermediate, or ancient grasslands) and plant-soil combinations (plants grown in "home" soil from their site of origin or "away" soil from other age groups). The box plot shows the median value and the first and third quartiles of the data. The whisker lines denote data range excluding outliers which extend beyond 1.5 times the interquartile range. Significant model parameters are presented in the figure with significant level indicators: $P < 0.05$, *; $P < 0.001$, ***. SRL, specific root length.

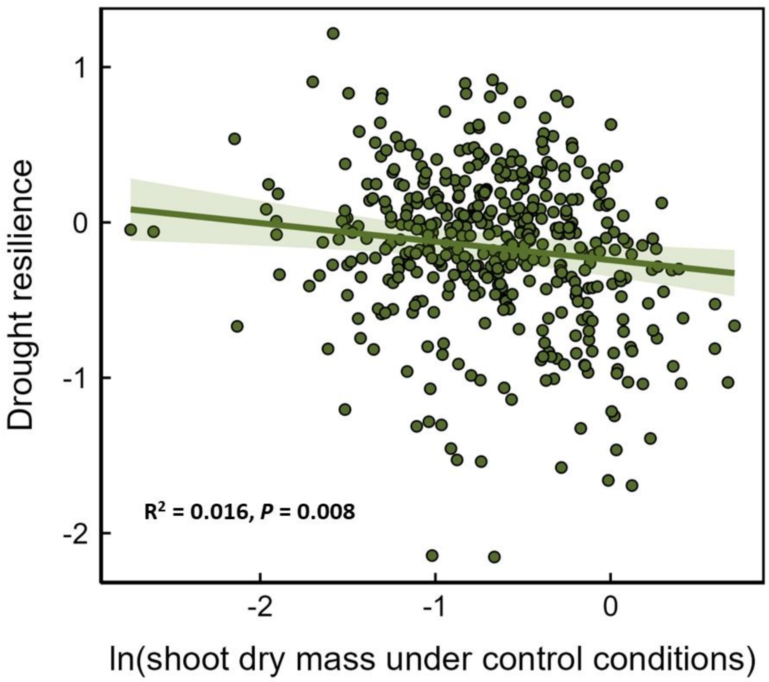

Figure S8 Relationship between drought resilience and shoot biomass under non-limiting water conditions across genotypes of *Briza media*. Drought resilience was calculated for each genotype as the natural log-transformed ratio of shoot dry mass in the drought treatment to that under control conditions. The dots represent individual observations. The solid line indicates the regression line, and shaded area displays 95% confidence interval of the fitted line. R^2 accounts for the proportion of variance explained by fixed effects alone.

Table S1 Soil abiotic properties of the grasslands. Data is presented as average value of the five sites at each grassland age group (young, intermediate, and ancient), and the standard deviation. Soil structure indicates the mass percentage of clay, silt, and sand in dry soil (<2 mm). The total organic carbon (TOC) and total nitrogen (total N) contents are presented as percentage of dry soil mass. The concentrations of nitrogen (ammonium-N and nitrate-N) and phosphorus (Olsen-P and total P) are presented in mg kg^{-1} of dry soil. Statistical significance (F, Df, and P-values) and effect size (R^2) of grassland age effects are presented.

		Young grassland		Intermediate grassland		Ancient grassland						
		**average**	**std**	**average**	**std**	**average**	**std**	**F**	**Df**	**P-value**	R^2	$R^2_{(adj.)}$
**soil texture**	**clay/%**	2.62	1.15	4.57	1.82	3.29	1.54	2.10	2	0.17	0.259	0.135
	**silt/%**	47.04	13.37	50.34	10.66	48.89	8.34	0.11	2	0.89	0.018	-0.145
	**sand/%**	50.34	14.46	45.09	11.55	47.82	9.83	0.24	2	0.79	0.038	-0.123
**pH**		7.09	0.17	6.70	0.25	6.77	0.07	**6.76**	**2**	**0.01**	**0.530**	**0.451**
**TOC**		4.37	0.98	4.31	1.05	4.48	0.58	0.10	2	0.90	0.017	-0.147
**Total N**		0.45	0.10	0.45	0.19	0.50	0.20	0.15	2	0.87	0.024	-0.139
**Ammonium-N**		6.08	3.82	4.87	1.41	4.42	0.95	0.51	2	0.61	0.078	-0.075
**Nitrate-N**		30.01	9.39	30.57	8.07	36.15	8.39	0.78	2	0.48	0.114	-0.033
**Olsen-P**		2.27	1.90	2.34	1.54	1.61	1.53	0.29	2	0.75	0.046	-0.113
**Total P**		34.11	18.32	22.42	13.89	21.14	17.54	0.92	2	0.43	0.132	-0.012

Table S2 Soil fungal communities in the grassland soils. Data is presented as average value of the five sites at each grassland age group (young, intermediate, and ancient) and the standard deviation. Parameters of linear models used to assess differences in fungal properties among grassland age groups are presented.

		**Young grasslands**		**Intermediate grasslands**		**Ancient grasslands**						
		**mean**	**sd**	**mean**	**sd**	**mean**	**sd**	**Df**	**F**	**P-value**	**R^2**	**$R^2_{(adj.)}$**
**Shannon index**	**Total fungi**	5.40	0.24	4.67	0.74	4.79	0.31	2	3.30	0.072	0.355	0.247
	**Putative pathogen**	3.55	0.48	3.74	0.42	3.81	0.17	2	0.62	0.556	0.093	-0.058
	**AM fungi**	3.21	0.23	1.88	0.97	1.78	0.72	**2**	**6.26**	**0.014**	**0.511**	**0.429**
**Relative abundance**	**Putative pathogen**	0.14	0.04	0.06	0.03	0.06	0.03	**2**	**8.26**	**0.006**	**0.579**	**0.509**
	**AM fungi**	0.005	0.004	0.001	0.001	0.0007	0.0005	**2**	**6.27**	**0.014**	**0.511**	**0.429**

Table S3 Summary of linear mixed effects models analyzing the effects of drought, plant-soil combination, plant population age group (*Briza media* genotypes originating from either young, intermediate, or ancient grasslands) and their interactions on CO_2 fluxes. For each factor, the F value, Df, Df.res), and *P* values are given. $R^2_{cond.}$ – conditional R^2, i.e. the proportion of variance explained by fixed and random effects. $R^2_{marg.}$ – marginal R^2, i.e. the proportion of variance explained by fixed effects alone. D, drought; P, plant population age group; H, plant-soil combination ("home" vs. "away").

		D	P	H	D:P	D:H	P:H	D:P:H	$R^2_{cond.}$	$R^2_{marg.}$
Photosynthetic rate at peak drought	F	54.31	0.03	0.48	4.60	0.55	1.84	0.60	0.943	0.749
	Df	1	2	1	2	1	2	2		
	Df.res	14.25	9.02	40.78	40.57	40.82	48.24	45.45		
	P	**<0.001**	0.968	0.494	**0.016**	0.462	0.170	0.554		
Ecosystem respiration at peak drought	F	52.15	1.41	0.17	3.46	0.01	0.53	1.10	0.517	0.364
	Df	1	2	1	2	1	2	2		
	Df.res	56.55	8.69	56.55	56.55	56.55	61.01	56.55		
	P	**<0.001**	0.295	0.680	**0.038**	0.923	0.594	0.339		
NEE at peak drought	F	50.53	0.35	0.34	3.00	0.35	1.43	0.83	0.928	0.748
	Df	1	2	1	2	1	2	2		
	Df.res	14.26	8.83	41.63	41.34	41.70	44.20	46.90		
	P	**<0.001**	0.715	0.562	0.061	0.558	0.250	0.443		
Photosynthetic rate at early recovery	F	17.52	0.02	1.20	0.70	2.73	0.73	0.09	0.725	0.306
	Df	1	2	1	2	1	2	2		
	Df.res	13.44	48.24	48.13	48.27	48.14	60.80	61.52		
	P	**0.001**	0.978	0.280	0.503	0.105	0.487	0.911		
Ecosystem respiration at early recovery	F	22.58	0.05	0.14	0.72	2.17	1.59	1.02	0.600	0.154
	Df	1	2	1	2	1	2	2		
	Df.res	56.15	9.61	56.15	56.15	56.15	64.13	56.15		
	P	**<0.001**	0.952	0.705	0.489	0.147	0.211	0.368		
NEE at early recovery	F	14.67	0.20	0.64	0.89	1.19	0.63	0.21	0.600	0.299
	Df	1	2	1	2	1	2	2		
	Df.res	13.28	8.01	41.20	41.68	41.09	51.36	54.80		
	P	**0.002**	0.826	0.428	0.417	0.283	0.538	0.815		
Photosynthetic rate at late recovery	F	20.03	0.73	1.22	0.65	2.85	0.18	2.03	0.698	0.233
	Df	1	2	1	2	1	2	2		
	Df.res	11.86	8.15	42.10	42.09	42.13	49.54	40.64		
	P	**<0.001**	0.510	0.275	0.528	0.099	0.838	0.145		
Ecosystem respiration at late recovery	F	17.31	0.08	2.10	2.50	0.66	0.58	1.97	0.744	0.091
	Df	1	2	1	2	1	2	2		
	Df.res	56.02	8.11	56.02	56.02	56.02	62.77	56.02		
	P	**<0.001**	0.926	0.153	0.091	0.420	0.562	0.149		
NEE at late recovery	F	12.67	0.35	0.59	1.62	2.92	0.00	1.72	0.573	0.206
	Df	1	2	1	2	1	2	2		
	Df.res	54.47	8.45	54.29	55.01	55.47	65.47	54.34		
	P	**<0.001**	0.714	0.447	0.207	0.093	0.997	0.188		

Table S4 Selection of the best predictors of drought response of CO_2 fluxes. Each selected variable was included in linear mixed models with interactions between drought treatment and plant-soil combination as fixed factors, and the sampling site was included as a random factor. The fit of linear mixed models was shown with Akaike Information Criterion (AIC) and coefficient of determination (R^2). The best predictors are shown in bold.

	AIC	R^2 (conditional)	R^2 (marginal)
**Photosynthetic rate at peak drought**			
Grassland soil age group	139.5	0.958	0.818
PCoA1 of total fungal composition	133.99	0.96	0.799
Pathogen abundance	**119.98**	**0.964**	**0.818**
PCoA2 of pathogen composition	134.8	0.957	0.822
AM fungi abundance	128.5	0.964	0.788
Shannon index of AM fungi	127.12	0.962	0.791
PCoA1 of AM fungal composition	135.43	0.958	0.803
**Photosynthetic rate at early recovery**			
Grassland soil age group	164.95	0.748	0.415
PCoA1 of total fungal composition	159.41	0.742	0.387
Pathogen abundance	**154.66**	**0.739**	**0.421**
PCoA2 of pathogen composition	159.13	0.738	0.424
AM fungi abundance	155.46	0.757	0.377
Shannon index of AM fungi	156.69	0.747	0.379
PCoA1 of AM fungal composition	157.96	0.744	0.398
**Photosynthetic rate at late recovery**			
Grassland soil age group	147.2	0.718	0.327
PCoA1 of total fungal composition	139.02	0.722	0.297
Pathogen abundance	142.54	0.706	0.269
PCoA2 of pathogen composition	**137.67**	**0.721**	**0.376**
AM fungi abundance	150.79	0.673	0.222
Shannon index of AM fungi	147.78	0.687	0.232
PCoA1 of AM fungal composition	141.27	0.707	0.283
**Ecosystem respiration at peak drought**			
Grassland soil age group	-74.44	0.486	0.334
PCoA1 of total fungal composition	-77.12	0.446	0.303
Pathogen abundance	-77.97	0.452	0.307
PCoA2 of pathogen composition	**-81.41**	**0.474**	**0.328**
AM fungi abundance	-76.31	0.438	0.301
Shannon index of AM fungi	-76.4	0.436	0.303

PCoA1 of AM fungal composition	-76.84	0.449	0.3
**Ecosystem respiration at early recovery**			
Grassland soil age group	-27.41	0.641	0.252
PCoA1 of total fungal composition	-30.68	0.619	0.213
Pathogen abundance	**-35.74**	**0.648**	**0.239**
PCoA2 of pathogen composition	-33.78	0.603	0.266
AM fungi abundance	-33.27	0.645	0.223
Shannon index of AM fungi	-29.33	0.627	0.179
PCoA1 of AM fungal composition	-32.49	0.631	0.216
**Ecosystem respiration at late recovery**			
Grassland soil age group	-42.67	0.724	0.166
PCoA1 of total fungal composition	-48.05	0.715	0.145
Pathogen abundance	-45.3	0.702	0.139
PCoA2 of pathogen composition	**-50.7**	**0.715**	**0.231**
AM fungi abundance	-41.08	0.689	0.102
Shannon index of AM fungi	-40.79	0.687	0.103
PCoA1 of AM fungal composition	-48.67	0.716	0.161
**Net ecosystem exchange at peak drought**			
Grassland soil age group	169.62	0.819	0.788
PCoA1 of total fungal composition	171.55	0.798	0.775
Pathogen abundance	**159.91**	**0.823**	**0.803**
PCoA2 of pathogen composition	162.01	0.818	0.798
AM fungi abundance	170.82	0.799	0.777
Shannon index of AM fungi	173.72	0.793	0.770
PCoA1 of AM fungal composition	170.23	0.801	0.779
**Net ecosystem exchange at early recovery**			
Grassland soil age group	167.37	0.61	0.369
PCoA1 of total fungal composition	161.32	0.602	0.343
Pathogen abundance	**155.98**	**0.601**	**0.387**
PCoA2 of pathogen composition	161.16	0.603	0.359
AM fungi abundance	157.43	0.611	0.357
Shannon index of AM fungi	158.06	0.6	0.357
PCoA1 of AM fungal composition	160.39	0.603	0.353
**Net ecosystem exchange at late recovery**			
Grassland soil age group	136.12	0.603	0.272
PCoA1 of total fungal composition	127.05	0.619	0.26
Pathogen abundance	129.37	0.6	0.231

PCoA2 of pathogen composition	**124.81**	**0.634**	**0.307**
AM fungi abundance	136.23	0.561	0.19
Shannon index of AM fungi	133.42	0.578	0.2
PCoA1 of AM fungal composition	129.24	0.597	0.239

Table S5 Summary of linear mixed effects models analyzing the main effects of drought, plant-soil combination, plant population age group (*Briza media* genotypes originating from either young, intermediate, or ancient grasslands) and their interactions on plant traits and AM fungal colonization in roots. For each factor, the F value, Df (degree of freedom), Df.res (residual degrees of freedom), and *P* values are given. $R^2_{cond.}$ – conditional R^2, i.e. the proportion of variance explained by fixed and random effects. $R^2_{marg.}$ – marginal R^2, i.e. the proportion of variance explained by fixed effects alone. LDMC, leaf dry matter content. LCC, leaf carbon content. LNC, leaf nitrogen content. Avg.D, average root diameter. RTD, root tissue density. RMF, root mass fraction. SRL, specific root length.

		D	P	H	D:P	D:H	P:H	D:P:H	$R^2_{cond.}$	$R^2_{marg.}$
Shoot biomass	F	11.48	0.61	5.13	0.26	0.70	0.86	1.00	0.598	0.122
	Df	1	2	1	2	1	2	2		
	Df.res	12.63	50.44	50.44	50.44	50.44	61.03	44.84		
	P	**0.005**	0.547	**0.028**	0.774	0.406	0.429	0.378		
ln (Height)	F	436.72	0.08	0.00	0.10	0.35	0.26	1.02	0.861	0.687
	Df	1	2	1	2	1	2	2		
	Df.res	56.15	10.13	56.15	56.15	56.15	61.78	56.15		
	P	**<0.001**	0.921	0.960	0.902	0.554	0.770	0.368		
LDMC	F	1.51	0.75	1.24	0.01	1.63	1.31	0.43	0.441	0.073
	Df	1	2	1	2	1	2	2		
	Df.res	56.20	8.18	56.20	56.20	56.20	68.15	56.20		
	P	0.224	0.501	0.270	0.990	0.207	0.277	0.651		
Sqrt (LCC)	F	0.01	1.18	0.15	1.99	0.06	1.06	0.60	0.16	0.103
	Df	1	2	1	2	1	2	2		
	Df.res	66	12	66	66	66	66	66		
	P	0.908	0.339	0.703	0.145	0.805	0.352	0.552		
ln (LNC)	F	13.46	1.95	0.71	0.08	0.53	0.32	0.44	0.478	0.124
	Df	1	2	1	2	1	2	2		
	Df.res	56.19	8.13	56.19	56.19	56.19	68.28	56.19		
	P	**<0.001**	0.204	0.403	0.920	0.468	0.726	0.647		
Root mass	F	0.08	1.94	2.55	1.69	6.56	0.06	0.97	0.704	0.062
	Df	1	2	1	2	1	2	2		
	Df.res	64.01	64.01	64.01	64.01	64.01	71.84	64.01		
	P	0.785	0.152	0.115	0.193	**0.013**	0.938	0.385		
SRL	F	5.29	1.39	2.09	4.58	0.02	0.04	0.32	0.619	0.096
	Df	1	2	1	2	1	2	2		
	Df.res	12.68	50.34	50.34	50.34	50.34	59.73	50.01		
	P	**0.039**	0.260	0.155	**0.015**	0.876	0.957	0.730		
Avg.D	F	2.09	0.05	10.07	1.58	0.63	2.50	1.14	0.615	0.104
	Df	1	2	1	2	1	2	2		
	Df.res	66.00	12.00	66.00	66.00	66.00	66.00	66		
	P	0.153	0.952	**0.002**	0.213	0.429	0.090	0.327		
RTD	F	43.30	0.57	1.73	0.51	0.64	0.68	0.25	0.468	0.302

	Df	1	2	1	2	1	2	2		
	Df.res	64.12	64.12	64.12	64.12	64.12	74.14	64.12		
	P	**<0.001**	0.570	0.193	0.604	0.427	0.510	0.779		
RMF	F	9.66	0.49	9.64	1.70	4.22	0.61	0.23	0.59	0.141
	Df	1	2	1	2	1	2	2		
	Df.res	64.02	64.02	64.02	64.02	64.02	76.09	64.02		
	P	**0.003**	0.614	**0.003**	0.192	**0.044**	0.547	0.796		
Hyphae	F	0.66	0.28	0.19	0.09	0.03	2.02	0.29	0.249	0.074
	Df	1	2	1	2	1	2	2		
	Df.res	12.61	50.60	50.60	50.60	50.60	59.47	45.47		
	P	0.432	0.758	0.662	0.911	0.870	0.142	0.749		
Arbuscules	F	0.09	0.84	0.68	1.60	1.30	0.21	0.72	0.266	0.081
	Df	1	2	1	2	1	2	2		
	Df.res	12.87	50.50	50.50	50.50	50.50	52.67	57.98		
	P	0.765	0.436	0.413	0.212	0.260	0.810	0.491		
Vesicles	F	1.43	3.10	0.31	0.17	0.04	0.80	0.06	0.41	0.078
	Df	1	2	1	2	1	2	2		
	Df.res	12.69	50.45	50.45	50.45	50.45	64.32	48.19		
	P	0.254	0.054	0.582	0.844	0.850	0.455	0.943		

Table S6 Selection of the best predictors of drought response of aboveground productivity. When shoot biomass was the response variable, each selected variable was included in a linear mixed-effects models with drought treatment, plant-soil combination, and their interactions as fixed factors. When drought resilience was the response variable, each selected variable was included in interaction with plant-soil combination as fixed factors. In both models, sampling site was included as a random factor. The fit of linear mixed models was shown with Akaike Information Criterion (AIC) and coefficient of determination (R^2). The best predictors are shown in bold.

	**AIC**	**R^2 (conditional)**	**R^2 (marginal)**
**Shoot biomass**			
Grassland soil age group	8.66	0.586	0.242
PCoA1 of total fungal composition	5.74	0.573	0.143
Pathogen abundance	**1.14**	**0.6**	**0.161**
PCoA2 of pathogen composition	**2.46**	**0.581**	**0.212**
AM fungi abundance	6.11	0.57	0.147
Shannon index of AM fungi	6.75	0.567	0.144
PCoA1 of AM fungal composition	3.65	0.586	0.149
**Drought resilience for mesocosm aboveground productivity**			
Grassland soil age group	28.36	0.119	0.006
PCoA1 of total fungal composition	28.24	0.04	-0.03
Pathogen abundance	**24.24**	**0.122**	**0.057**
PCoA2 of pathogen composition	**23.69**	**0.132**	**0.069**
AM fungi abundance	27.23	0.061	-0.007
Shannon index of AM fungi	28.05	0.044	-0.026
PCoA1 of AM fungal composition	28.71	0.03	-0.041

Table S7 The list of operational taxonomic units (OTUs) that significantly correlated with the second axis of Principal Coordinates Analysis (PCoA) of putative fungal pathogen composition in 15 soils used in the study, and the correlation between the relative abundance of the OTUs and plant drought resilience. The direction (negative or positive), strength, and significance of the correlation between OTUs and the second PCoA axis are shown. The correlation coefficient and significance of the association between the relative abundance of each OTU and plant drought resilience (calculated as the natural log-transformed ratio of plant shoot biomass in drought versus control conditions) are displayed, with significant correlations highlighted in bold.

OUT ID		PCoA2		Drought resilience		Family	Genus	Species
		r^2	*P*	*r*	*P*			
OTU41607	-	0.32	0.030	**-0.33**	**0.028**	Nectriaceae	Fusarium	Fusarium algeriense
OTU39350	+	0.28	0.042	0.08	0.583	Nectriaceae	Ilyonectria	Ilyonectria mors-panacis
OTU49573	-	0.31	0.025	**-0.40**	**0.006**	Didymellaceae	Epicoccum	Epicoccum pimprinum
OTU78647	-	0.52	0.003	-0.26	0.080	Phaeosphaeriaceae	Paraphoma	Paraphoma ledniceana
OTU49723	-	0.41	0.014	-0.26	0.086	Mycosphaerellaceae	Cercospora	
OTU40190	+	0.26	0.046	0.11	0.487	Nectriaceae	Fusarium	
OTU67880	-	0.31	0.042	-0.21	0.159	Ploettnerulaceae	Mycochaetophora	
OTU77760	-	0.37	0.018	-0.21	0.158	Phaeosphaeriaceae	Paraphoma	Paraphoma ledniceana
OTU61355	-	0.42	0.009	-0.21	0.163	Melanommataceae	Petrakia	Petrakia sp
OTU50498	+	0.38	0.014	0.26	0.083	Didymellaceae	Calophoma	Calophoma rosae
OTU10962	+	0.26	0.047	-0.07	0.646	Ceratobasidiaceae	Ceratobasidium	Ceratobasidium ramicola
OTU51359	+	0.36	0.022	**0.38**	**0.009**	Phaeosphaeriaceae	Ophiosphaerella	Ophiosphaerella sp
OTU14519	+	0.38	0.013	0.21	0.162	Rickenellaceae	Rickenella	Rickenella swartzii
OTU60660	+	0.34	0.033	**0.34**	**0.021**	Melanommataceae	Petrakia	Petrakia sp
OTU71310	-	0.36	0.019	-0.17	0.253	Ploettnerulaceae	Mycochaetophora	Mycochaetophora gentianae
OTU55618	+	0.35	0.028	0.21	0.162	Didymellaceae	Phoma	
OTU47327	-	0.32	0.027	-0.27	0.076	Glomerellaceae	Colletotrichum	Colletotrichum tanaceti
OTU40960	-	0.31	0.029	**-0.33**	**0.025**	Nectriaceae	Fusarium	
OTU53486	-	0.33	0.020	**-0.44**	**0.003**	Didymellaceae	Ascochyta	Ascochyta medicaginicola var. macrospora
OTU46958	+	0.37	0.016	0.17	0.256	Phaeosphaeriaceae	Setophoma	Setophoma sp
OTU55410	+	0.47	0.004	0.22	0.154	Massarinaceae	Stagonospora	Stagonospora forlicesenensis
OTU41069	-	0.50	0.006	-0.23	0.122	Nectriaceae	Fusarium	
OTU41026	-	0.30	0.037	-0.28	0.063	Nectriaceae	Fusarium	
OTU68084	-	0.30	0.024	-0.10	0.497	Ploettnerulaceae	Mycochaetophora	
OTU40331	-	0.26	0.044	-0.24	0.114	Nectriaceae	Fusarium	
OTU38572	+	0.46	0.008	0.24	0.116	Microdochiaceae	Microdochium	Microdochium trichocladiopsis
OTU41634	-	0.40	0.006	-0.15	0.331	Nectriaceae	Fusarium	
OTU41613	-	0.30	0.034	**-0.35**	**0.019**	Nectriaceae	Fusarium	
OTU64271	+	0.54	0.001	0.15	0.326	Melanommataceae	Pseudodidymella	
OTU41083	-	0.24	0.043	-0.20	0.183	Nectriaceae	Fusarium	
OTU57849	+	0.52	0.003	**0.32**	**0.030**	Massarinaceae	Stagonospora	Stagonospora tainanensis
OTU41962	-	0.47	0.007	-0.19	0.212	Nectriaceae	Fusarium	
OTU41637	-	0.29	0.046	-0.09	0.551	Nectriaceae	Fusarium	
OTU61594	-	0.30	0.028	-0.24	0.105	Nectriaceae	Fusarium	Fusarium oxysporum

Method S1 Characterization of field-collected soil

The soil collected from the field was characterized in terms of texture (sand, silt, and clay content), pH, total organic carbon, total nitrogen, ammonium-N, nitrate-N, Olsen-phosphorus, and total phosphorus. Soil texture was determined by a laser diffraction method. The soil pH was measured using a pH meter, with a soil-to-water ratio of 1:2.5. Total organic carbon, determined after the removal of inorganic carbon through HCl-fumigation (Harris *et al.* 2001), and total nitrogen were analyzed with an elemental analyzer. Olsen-phosphorus was extracted from 2 g of fresh soil (<2 mm) using 40 ml of 0.5 M $NaHCO_3$ solution (Olsen 1954), while the total soil phosphorus was extracted with 1 M HCl (soil-to-solution ratio 1:100) after calcination at 450°C (Wuenscher *et al.* 2015). The extracted Olsen-phosphorus and total phosphorus were then determined using the ammonium molybdate-malachite green assay (D'Angelo *et al.* 2001). The photometric absorbance of the color reactions was then analyzed using a Microplate Reader (Infinite M200, TECAN, Switzerland). Ammonium and nitrate were extracted from 2 g fresh soil (passed through a 2 mm sieve and measured at the start of the mesocosm experiment) using 15 mL of 0.5 M K_2SO_4 solution. The extracted ammonium and nitrate were then quantified using colorimetric methods: the Berthelot reaction for ammonium, and the acidic Griess reaction (after VCl_3 reduction) for nitrate (Hood-Nowotny *et al.* 2010).

Method S2 Measurement of plant traits

On day 20 of drought recovery, plants were harvested and plant traits were measured. For LDMC measurement, three to five leaf segments (5–10 cm in length) were excised from the median portion of the leaf blade for each *Briza media* genotype. These segments were placed vertically in sealed polyethylene bags containing a small volume of water and maintained in darkness at 4°C for 24 h to achieve full hydration. After hydration, fresh mass was determined. The leaves were then oven-dried at 40°C until reaching constant mass. LDMC was calculated as the ratio of leaf dry mass to fully hydrated fresh mass.

References

1.
D'Angelo, E., Crutchfield, J. & Vandiviere, M. (2001). Rapid, sensitive, microscale determination of phosphate in water and soil. *Journal of environmental quality*, 30, 2206-2209.
2.
Harris, D., Horwáth, W.R. & Van Kessel, C. (2001). Acid fumigation of soils to remove carbonates prior to total organic carbon or carbon-13 isotopic analysis. *Soil Science Society of America Journal*, 65, 1853-1856.
3.
Hood-Nowotny, R., Umana, N.H.-N., Inselbacher, E., Oswald-Lachouani, P. & Wanek, W. (2010). Alternative methods for measuring inorganic, organic, and total dissolved nitrogen in soil. *Soil Science Society of America Journal*, 74, 1018-1027.
4.
Olsen, S.R. (1954). *Estimation of available phosphorus in soils by extraction with sodium bicarbonate*. US Department of Agriculture.
5.
Wuenscher, R., Unterfrauner, H., Peticzka, R. & Zehetner, F. (2015). A comparison of 14 soil phosphorus extraction methods applied to 50 agricultural soils from Central Europe. *Plant, Soil and Environment*, 61, 86-96.
